# Valorization of birch tops and branches containing mechanically inseparable wood and bark

**DOI:** 10.1039/d6gc01905f

**Published:** 2026-06-01

**Authors:** Lala Ramazanova, Eliise Tammekivi, Anneli Kruve, Joseph S. M. Samec

**Affiliations:** a Department of Chemistry, Stockholm University, Arrhenius Laboratories 10691 Stockholm Sweden joseph.samec@su.se

## Abstract

If valorization of tops and branches would be realized, there is potential to increase the overall yield from forestry by 50%, which would impact land use change and climate change considerably. Currently, these residues are either incinerated or left to decay in the forests. The main challenge of valorization of tops and branches is their composition: comprising both wood and bark that are mechanically inseparable. Herein, we propose a chemical approach to valorize all components of both wood and bark of tops and branches without prior mechanical separation. By applying a sequential fractionation sequence involving two different catalytic steps, all different parts of the tops and branches have been valorized into well-defined streams and an intact cellulose-rich pulp. It was found that the sequence of processes was important for the overall outcome. We hope that this first report of the valorization of tops and branches will inspire further studies to unlock the full potential of this challenging yet abundant feedstock.

## Abstract 2

Valorisation of both bark and wood components of tops and branches without prior mechanical separation was realized.

## Graphical abstract

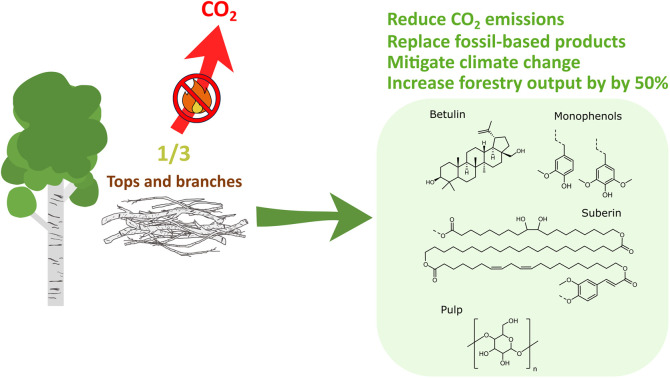

**Green foundation**

1. This work advances green chemistry by demonstrating that a challenging feedstock that is currently burnt can be valorized.

2. By using purely green chemical technologies including two catalytic steps, both bark and wood of tops and branches could be valorized into different products: 90% yield of extractives, mainly betulin; 75% of maximum yield of monophenols from lignin; and 67% yield of suberin, without prior mechanical separation.

3. By developing a flow-through process, solvents could potentially be recirculated before distillation to save solvent and energy.

## Introduction

**Fig. 1:**
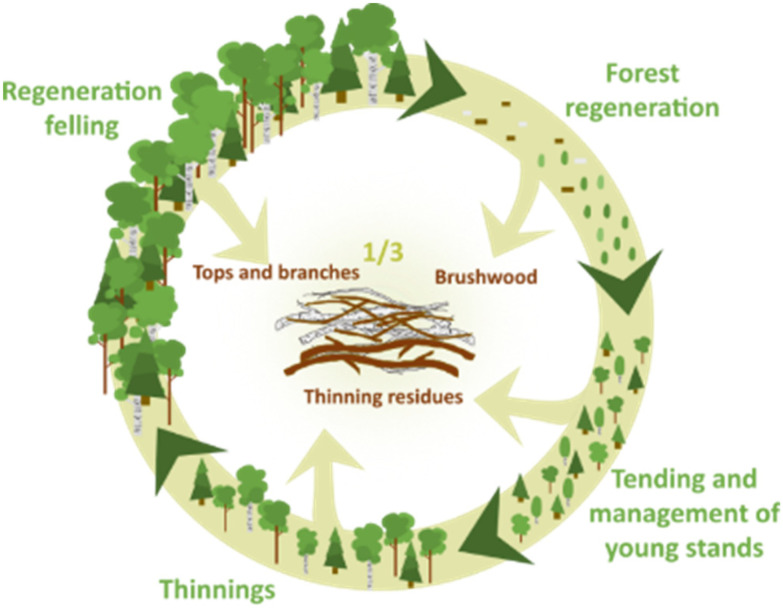
Forestry cycle and generated residues comprising both bark and wood that are not mechanically separable.

With the continual growth of the global population,^1^ the demand for resources is predicted to rise during the 21st century. Our main source for chemicals and materials is currently crude oil, which is expected to be depleted within 40 years.^2^ To substitute crude oil with biomass to cover our demands is not trivial.^3^ Deforestation, including land use change – embracing also the transformation of natural forests to plantation forests – negatively affects land use, including biodiversity loss.^4^ In addition, forests play a crucial role in the mitigation of climate change by storing carbon and reducing emissions associated with deforestation and forest degradation.^5^ A strategy to reduce deforestation, which is expected to increase over the coming decades,^6^ is to utilize the currently available biomass more efficiently. Exploiting forest residues has the potential to increase yields from existing forestry by 50% without utilizing new forest areas and thus aid in addressing the challenges described above. Forest residues (excluding roots) account for about 1/3 of total harvested biomass^7^ and are primarily composed of brushwood, which is usually removed to help seedlings grow; residues remaining after forest thinning, which is carried out to select the most competitive trees and reduce overall competition for nutrition and sunlight in order to maximize growth of the remaining stands; and tops and branches left after the final harvesting (see Fig. 1).^8,9^ Tops and branches are currently incinerated, as it is difficult to debark thin branches, where the bark content is higher than in other parts of the tree. Moreover, bark contains several components beyond the typical biomass constituents, namely cellulose, lignin, and hemicellulose.^10^ These additional components including suberin, tannins, and significantly higher levels of extractives and ash,^11,12^ make valorization of bark challenging due to a lack of effective fractionation procedures. While a portion of the tops and branches should remain in forests to support recarbonization and biodiversity,^13,14^ leaving excessive amounts in forests to decay results in greenhouse gas (GHG) emissions.^15^ Furthermore, the extractives’ rich tops and branches release compounds that poison the soil, termed allelopathy.^16^ Currently, the sustainable outtake of tops and branches from forests is being incinerated.^17^ While incineration is often considered carbon neutral, it is not climate neutral, thus affecting the global warming potential (GWP). The current discourse emphasizes non-carbon heat and power technologies such as wind, water and solar power.

Numerous studies focus on the fractionation of wood. Among recent fractionation strategies aimed at lignin valorization, the “lignin-first” approaches, where lignin is liberated from the plant cell walls while avoiding condensation reactions occurring during fractionation, have gained considerable attention.^18–24^ One of these approaches is reductive catalytic fractionation (RCF), a solvent-based extraction of lignin in the presence of a transition metal catalyst under a hydrogen atmosphere or in the presence of a hydrogen donor that yields a viscous lignin oil comprising monophenols.^18,20,21^ These methods have successfully been applied to hardwoods with high (up to 70%) β-ether bond content, depolymerizing lignin into monophenols with yields up to 50%.^25–27^ In comparison with wood, considerably less research has focused on the catalytic fractionation of bark.^28–30^ Within the limited studies available, bark fractionation has primarily focused either on the depolymerization of suberin under basic conditions^31–34^ or the extraction of valuable compounds other than suberin.^35–39^ Suberin is an insoluble hydrophobic aliphatic biopolymer with a low oxygen content, making it a potential feedstock for biofuel^11^ or a precursor for the preparation of aliphatic biopolyesters.^40^ The valorization of bark alone has the potential to increase the value of pulp and timber; however, it would not be sufficient for tops and branches.

No studies have addressed the combined fractionation of both wood and bark, which would open up avenues to tops and branches, with the potential to increase forestry output by 50%. In this study, we hypothesized and later even successfully demonstrated that tops and branches, that is, biomass comprising non-separable mixtures of bark and wood, can be valorized using a sequential chemical fractionation–depolymerization approach, removing different fractions selectively into well-defined streams susceptible to further downstream processing.

## Results and discussion

The samples of birch (*Betula pendula*) wood and bark were acquired from Björkträ Timber AB in Sweden. The tops and branches sample comprising a value of wood : bark 7 : 3 was used in this study. This ratio was based on literature studies,^41^ where samples were collected and the bark and wood were separated and gravimetrically analyzed.

The chemical composition of a representative top-and-branch sample containing a 7 : 3 mixture of wood to bark was performed and in parallel, both wood and bark were additionally analyzed separately. Tops and branches were first dried and the moisture content (see the SI, S2) in the mixture was determined to be 4.4 wt%, which is close to the moisture content in both wood (4.2 wt%) and bark (2.8 wt%). The ash content of the top-and-branch mixture was determined by combustion of organics to be 0.32 wt%, which is close to that of wood (0.36 wt%) and bark (0.31 wt%).

**Fig. 2:**
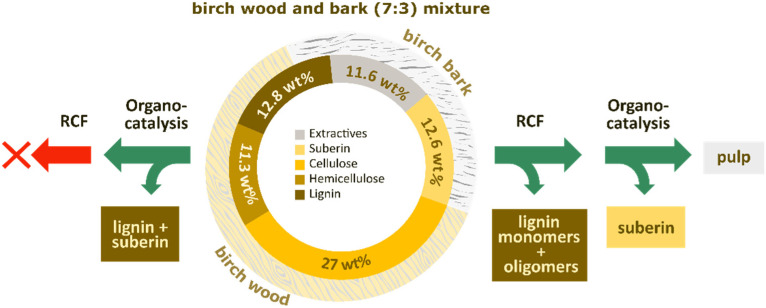
Composition of the 7 : 3 wood-to-bark mixture and comparison of approaches for valorization of birch tops and branches.

Extractive content in the top-and-branch mixture determined by Soxhlet extraction with EtOH was 11.6 wt%, where most of the extractives stemmed from the bark part (33 wt%) and only 3 wt% was found in the wood (see the SI, S3). Suberin content in the tops and branches was analyzed through basic hydrolysis and determined to be 12.6 wt%, which originated solely from the bark (42 wt%) (see the SI, S4). Carbohydrate and acid-insoluble lignin (AIL) contents were determined using the Klason^42^ method (see the SI, S8). Cellulose content was determined to be 27 wt%, mostly originating from wood (44 wt%) with only a small fraction derived from bark (1.4 wt%). Hemicellulose content was determined to be 11.3 wt%, also mainly originating from the woody part (20 wt%) and with minor contributions from bark (2.5 wt%). AIL content was determined to be 12.8 wt%, of which 70% originated from wood (17 wt%) and 30% from bark (13 wt%) (see Fig. 2).

To enable the valorization of both bark and wood components in the mixture, two different approaches were envisioned (see Fig. 2). The first, reductive catalytic fractionation (RCF), has been shown to be effective on woody parts of hardwoods and can yield around 50 wt% monophenols based on lignin content. However, RCF conditions are not efficient for valorization of suberin. The other approach, organo-catalysis, has previously been successfully applied in our group for the depolymerization of suberin from birch bark; however, it is not efficient for lignin.^43^ We screened a two-process sequence strategy: in the first approach, organo-catalysis was applied initially to extract and to some extent depolymerize suberin from bark, followed by RCF of the remaining residue to convert lignin to monophenols and liberate cellulose. Although the organocatalysis step effectively extracted and, to some extent, depolymerized suberin along with a portion of lignin, the subsequent RCF did not yield any monophenols. Most likely, the lignin underwent recondensation reactions under the applied organocatalysis, known to occur under organosolv conditions, which shuts down the RCF. To support the proposal of lignin recondensation occurring during the organocatalysis-first step, we performed a control experiment where thioacidolysis was performed after the organocatalysis step of the extracted tops and branches. Interestingly, no lignin monomers were detected, demonstrating that organocatalysis disrupted the alkyl aryl ether linkages (including β-O-4) responsible for releasing monomers (see the SI, S9, Fig. S17). This suggests that the lignin underwent recondensation reactions during the organocatalysis step. However, the reverse sequence, RCF followed by organocatalysis of the solid residue, provided successful results. Gratifyingly, the organocatalysis step was not affected by the RCF. Thus, we decided to apply RCF before the organocatalysis.

**Fig. 3:**
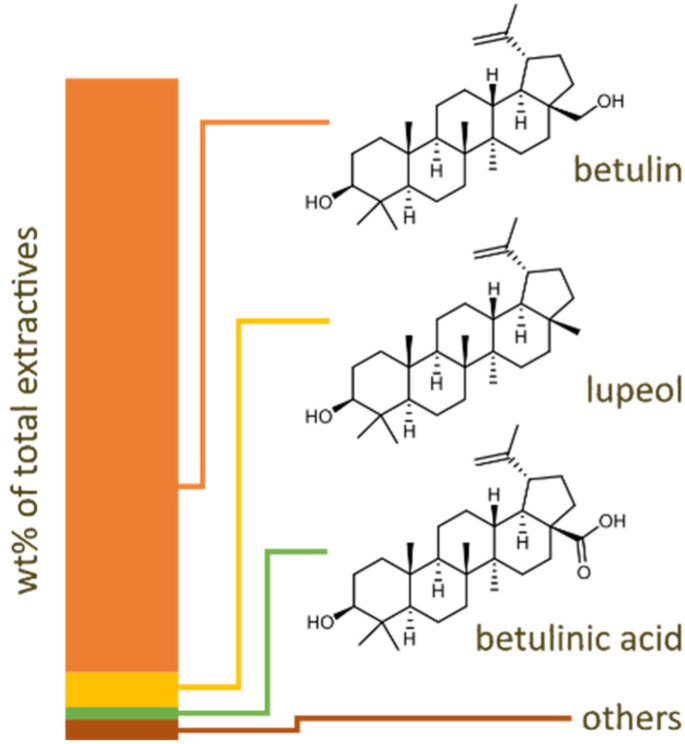
Distribution of lipophilic extractives in the product mixture.

First, the extractives were removed from the top-and-branch mixture using EtOH in a Soxhlet reactor for 12 hours. This yielded 11.6 wt% of extractives, which corresponds to 90% of the theoretical maximum yield and comprised mainly triterpenoids. For the analysis of the extractives, liquid chromatography high-resolution mass spectrometry (LC-HRMS) was used. Commercial standards of betulin, lupeol, betulinic acid, and ferulic acid were used for the identification and quantification. These compounds were identified in the extractive fraction based on LC retention time and high-resolution mass spectral data matching. Quantification was performed by external calibration, using individual calibration curves constructed from measuring a series of calibration solutions prepared from the corresponding standards. The details of the LC-HRMS analysis are presented in the SI (see SI S3). Quantification of the main compounds found in the mixture showed betulin (89.7 wt%), followed by lupeol (5.3 wt%), betulinic acid (1.8 wt%), and a trace amount of ferulic acid. These results align with the composition reported in the literature^44^ (see Fig. 3).

**Fig. 4:**
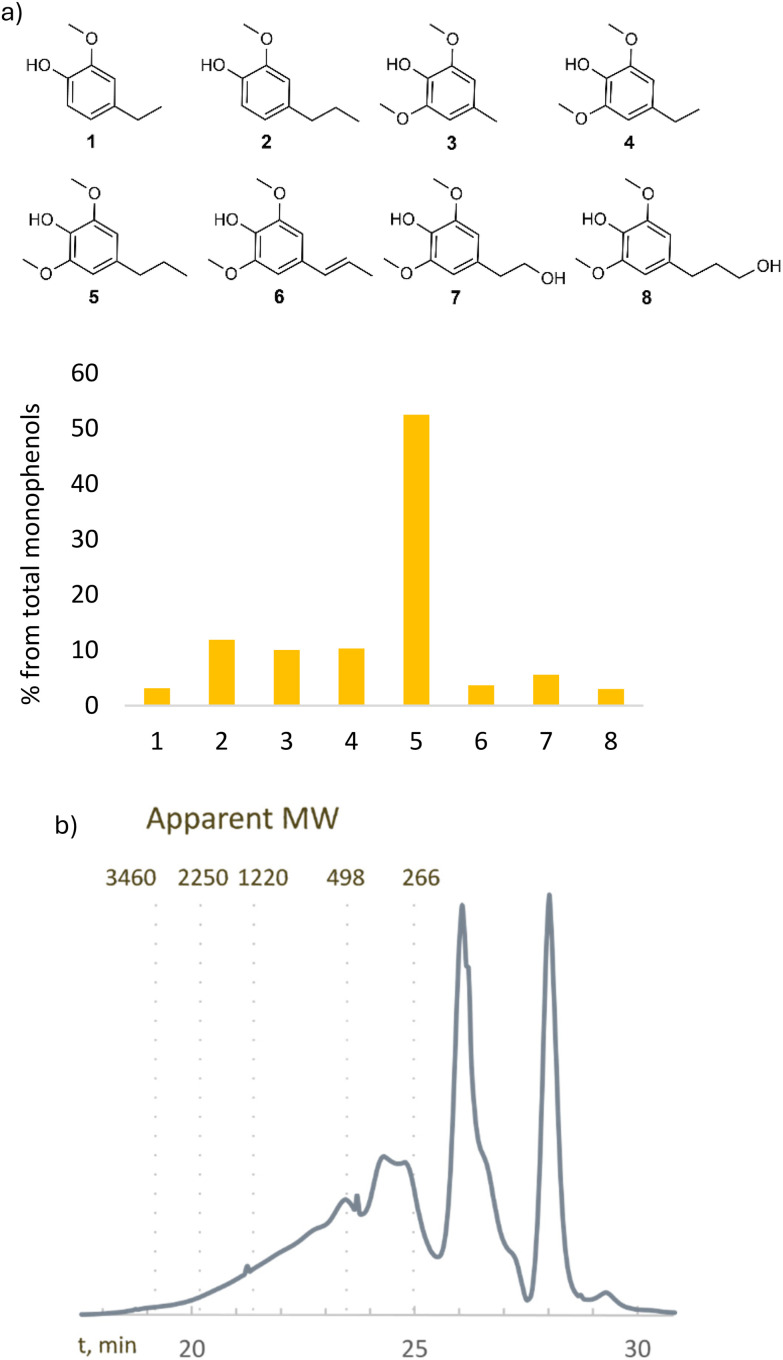
Distribution of monophenols (a) and molecular weight distribution (b) of the RCF product mixture.

RCF of the extracted wood and bark mixture in the presence of a Pd/C catalyst and formic acid was performed in a stainless-steel batch reactor at 200 °C for 4 hours (see the SI, S5). GC-MS was used for the qualitative analysis, while GC-FID using tetracosane as an internal standard was used to quantify the monophenols. RCF gave 75% yield of monophenols with respect to the theoretical maximum yield based on the β-ether content determined using thioacidolysis. This corresponds to 19 wt% of the lignin and 3% of the original biomass (see Fig. 4a). To gain more insights into the low yield – where the theoretical maximum yield was around 45% yield – we performed RCF on wood and bark separately. Although RCF on wood gave a yield close to the theoretical maximum yield of monophenols, the RCF on bark gave no yield of monophenols. Thus, RCF is only efficient for depolymerizing the lignin in the wood and this explains the lower yield of monophenols with respect to the total amount of lignin. The RCF product mixture was also examined for the presence of higher-molecular weight compounds. Size exclusion chromatography of the product mixture (see Fig. 4b) revealed the presence of compounds with apparent molecular weights above 266 Da, corresponding to various-sized lignin polymers and oligomers.

The resulting pulp was analyzed using the Klason method, which revealed a carbohydrate composition of 22.2 wt% of cellulose and 3 wt% of AIL, the latter stemming from the lignin in the bark part. The observed 18% loss of cellulose during RCF can be explained by its partial solubilization and carbohydrate transfer to the pulping liquor, which was not analysed for carbohydrate content. The analysis did not detect any hemicellulose in the solid residue after RCF either. Previous studies from our group have demonstrated that hemicellulose is solubilized under the applied conditions and acts as an internal hydrogen donor during lignin depolymerization.^45^

**Fig. 5:**
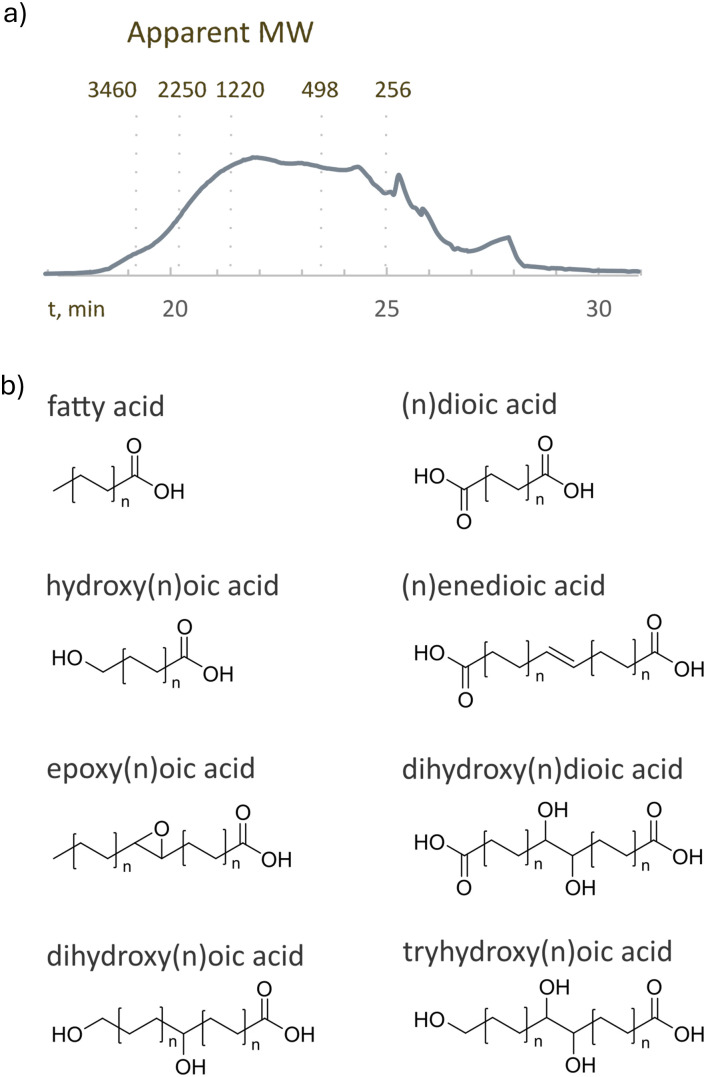
Molecular weight distribution of depolymerized suberin (a) and composition of monomeric fatty acids from the hydrolysed suberin mixture (b).

The remaining solid residue was subjected to organo-catalysis in the presence of Et_3_N using MeOH/H_2_O as the solvent (see the SI, S6). The reaction yielded 67% of the theoretical maximum yield of fatty acids and oligomers from the depolymerization of suberin (see Fig. 5a). This corresponds to an overall yield from the original biomass of 8.4%. This yield can be explained by the complex structure of suberin, where long-chain fatty acids are connected to phenolic compounds by ester bonds,^46^ and 2/3 of the positions of the polyaromatic domains are most susceptible to depolymerization (phenolic and allylic OH). Qualitative analysis of the hydrolysed suberin mixture using LC-HRMS (see the SI, S7) enabled the determination of its monomeric composition. The tentative identification of the hydrolyzed fatty acids was performed using SIRIUS,^47^ where structural candidates are proposed by matching the measured spectral data against known molecular formulae and structures. The elution order of fatty acid derivatives within the same group, differing only in carbon number, was compared to expected trends to support identification (see the SI, Fig. S14).

According to the analysis, the mixture is mainly composed of long-chain ω-hydroxycarboxylic and α, ω-dicarboxylic acids with the carbon chain lengths from C16 to C24 (see Fig. 5b). The presence of C16–24 monomers is consistent with previously reported suberin compositions. The peak area was the largest for octadecanoic acid (C_18_H_36_O_2_), docosanedioic acid (C_22_H_42_O_4_), hydroxydocosanoic acid (C_22_H_44_O_3_), octadecenedioic acid (C_18_H_32_O_4_), epoxyoctadecanoic acid (C_18_H_34_O_3_), dihydroxyoctadecanedioic acid (C_18_H_34_O_6_), dihydroxyhexadecanoic acid (C_16_H_32_O_4_), dihydroxyoctadecenoic acid (C_18_H_34_O_4_), trihydroxyoctadecanoic acid (C_18_H_36_O_5_), and trihydroxyeicosanoic acid (C_20_H_38_O_5_) in their respective compound class. This can be used as a proxy for concentration distribution within the respective compound class, assuming minimal impact of carbon number on the responsiveness of the compounds. The obtained product mixture was analyzed using gel permeation chromatography, which showed the presence of different molecular weight free fatty acids with different chain lengths and higher molecular weight oligomers, resulting from partial depolymerization of suberin (see Fig. 5a).

**Fig. 6:**
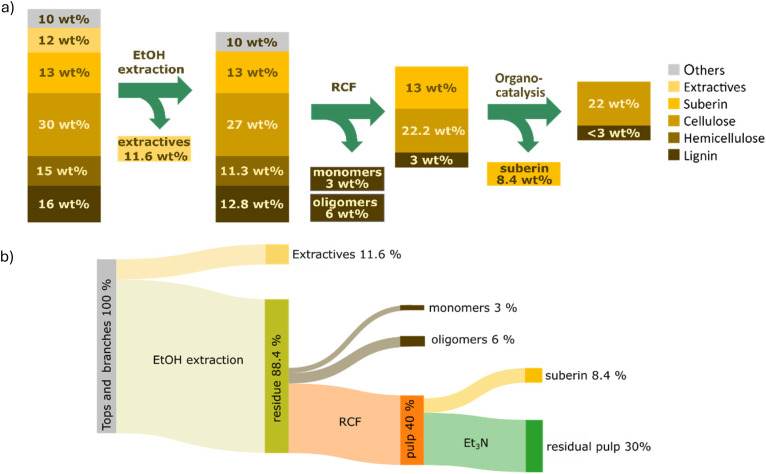
Sequential fractionation of the extracted wood and bark mixture (a) and mass balance of the entire sequence (b).

The remaining pulp was analyzed with respect to carbohydrate and lignin content, which revealed the presence of small amounts of lignin (< 3 wt%) and cellulose (22 wt%) from the original biomass (see Fig. 6a). Thus, the overall yield of cellulose from the initial 7 : 3 mixture of wood and bark was 73%. The transformation of 1 kg of tops and branches would give (see Fig. 6b) 116 g of extractives with a high content of betulin that has shown pharmaceutically interesting properties, such as cytotoxicity^48^ against a variety of tumor cells, 30 g of highly desirable monophenols and 60 g of phenolic oligomers that can be used to substitute phenol in resins.^49^ 84 g of suberin-derived fatty acids, which have the potential to substitute crop-based fatty acids, is generated^50^ in addition to 300 g of high-cellulose content pulp that can be used in various applications such as dissolving pulp or fermentation to ethanol.^14^ Birch is a major species in the Baltic region and is predicted to increase in importance due to spruce bark beetle spread and an increased demand for dissolving-grade pulp for textiles.

Currently, around 6 M tons of birch are harvested every year, generating 3 M tons of tops and branches, only in the Nordic and Baltic regions.^51^ This is an underestimation, as birch is also harvested during maintenance of softwood stands. By only calculating the 6 M tons, valorization would give 0.6 M tons of betulin, 0.15 M tons of monophenols, 0.3 M tons of phenol substitute, 0.42 M tons of fatty acids and 1.5 M tons of pulp. To put these numbers in perspective: Europe currently uses around 1.4 M tons of phenolic resins;^52^ current crude tall oil production capacity in the Nordic–Baltic region is 0.65 M tons per year;^53^ and rapeseed oil production is around 10 M tons.^54^ Dissolving pulp production in the same region is around 1 M ton.^55^ Thus, by valorizing this side stream, it has the potential to supply a large portion of phenols, increase the production of fatty acids by more than 50%, and increase the pulp production for textiles by 150%. It is noteworthy that the increase in production of these high-value products would not affect land use negatively. Furthermore, by avoiding incinerating this raw material, carbon dioxide emissions into the atmosphere are reduced without accounting for marginal effects for substitutions of resins, fatty acids and pulp.

## Conclusions

Sequential fractionation has successfully been applied to valorize tops and branches without the need to mechanically debark their woody parts. Lipophilic extractives such as terpenoids were efficiently removed. RCF allowed the conversion of lignin from the woody part of the mixture to monophenols, thereby increasing the value compared to its conventional use as a source of heat. Organo-catalysis further yielded suberin, a promising feedstock to produce bio-based polyesters or biofuels. The combination of extraction with these two approaches demonstrates an effective strategy to valorize this underutilized biomass and enable the production of compounds that are traditionally derived from fossil resources. It is important to note that the sequence of processes is important for upgrading both lignin and suberin. Upgrading an underutilized forestry residue would increase resource efficiency and generate additional revenue streams for the forestry sector while reducing CO_2_ emissions into the atmosphere. From a Nordic–Baltic perspective, valorization of tops and branches has the potential to supply current demands of tall oil and dissolving-grade pulp for textiles and additionally provide high-value extractives, monomeric aromatic substances and phenols: all without affecting land use. A direct consequence of valorizing the forest residues instead of incineration would be a reduction in carbon dioxide released into the atmosphere. Thus, there is notable potential in the valorization of tops and branches. However, to realize this, it is important to scale up the production, preferably in continuous flow, to monitor mass- and energy balances and to ascertain that the environmental and economic sustainability are favorable. Also, this technology is of interest to forest owners, the chemical industry, and national governments that could become both more geopolitically resilient and more sustainable.

## Conflicts of interest

There are no conflicts to declare.

## Supplementary Material

GC-OLF-D6GC01905F-s001

## Data Availability

All data are included in the supplementary information (SI). Any other data will be made available on request from the authors. Supplementary information is available. See DOI: https://doi.org/10.1039/d6gc01905f.
