# Supplementary material for: Valorization of birch tops and branches containing mechanically inseparable wood and bark
Source: Green Chem. 2026 Jun 1. Online ahead of print. doi: 10.1039/d6gc01905f (PMC13224099; doi:10.1039/d6gc01905f)
Supplement: GC-OLF-D6GC01905F-s001 [file GC-OLF-D6GC01905F-s001.pdf]

Supplementary Information (SI) for Green Chemistry.
This journal is © The Royal Society of Chemistry 2026

Supporting information

Valorization of birch tops and branches containing mechanically inseparable wood and bark

Lala Ramazanova[a], Eliise Tammekivi[a], Anneli Kruve[a] and Joseph S. M. Samec[a*]

[a]Department of Chemistry, Stockholm university, Arrhenius laboratories, 10691 Stockholm, Sweden

Corresponding Author

Joseph S. M. Samec, email: joseph.samec@su.se

S.1. General considerations

All chemicals were purchased from Fischer chemicals, CCS Healthcare AB Sweden, Sigma-Aldrich, Honeywell, and VWR chemicals, and used as received.

S.2. Determination of the moisture content

To determine the moisture content of biomass, raw sample of biomass was dried in an oven at 60 ºC for at least 12 h (overnight). The obtained weight loss corresponded to the moisture in biomass.

S.3. Pretreatment of biomass and LC-HRMS identification and quantification of the bark-wood extractives

In a typical extraction procedure, the biomass (raw birch wood and bark) was dried overnight at 60 ºC. To ensure the constant ratio of bark to wood (3:7) the biomass was dried separately. The dry biomass was weighed separately into a Soxlet thimble and mixed with a spatula. The extraction with EtOH was conducted overnight (12-14 h) at 100 ºC. The remaining biomass was dried in the oven at 60 ºC for 12 h and weighed to yield an extractive-free biomass (90 wt% of the initial biomass).

The remaining solvent was evaporated under reduced pressure to yield a brown solid lipophilic extractive in 11.6 wt% yield. The extractives were further analysed LC-HRMS.

Experimental parameters

The LC-HRMS experiments were performed on a Dionex UltiMate 3000 ultra-high performance (UHP)LC system hyphenated to a Q Exactive HF Orbitrap HRMS, both from Thermo Fisher Scientific (Bremen, Germany). For LC separation, a Kinetex F5 Core-Shell column (1.7 µm, 2.1 mm × 150 mm) from Phenomenex (Torrance, CA, USA) was used, with the following conditions: flow rate 0.30 mL/min, injection volume 5 µL, and oven temperature 40 °C. When the experiments were performed in electrospray positive ionisation mode (ESI+), the mobile phases were water (A) and methanol (B), both acidified with 0.1% formic acid (≥99% from VWR, Leuven, Belgium). For the negative mode (ESI-), the same solvents were used, but containing 2 mM ammonium acetate (≥99.99% from Sigma-Aldrich, Darmstadt, Germany). Gradient elution started at 60% of B, the content of B increased to 99% in 10 min and was held constant for 4 min, before it was decreased back to 60% B in 0.1 min. Equilibration time was 2.9 min, and the total run time was 17 min.

For the HRMS, the following parameters were used: spray voltage 3 kV (ESI-) or 4 kV (ESI+), capillary temperature 275 °C (ESI-) or 350 °C (ESI+), aux gas heater temperature 275 °C (ESI-) or 320 °C (ESI+), aux gas flow rate 5 arbitrary units (AU), sheath gas flow rate 30 AU, and RF lens 50% (ESI-) or 55% (ESI+). The aim was to achieve a peak intensity of at least 1×10^7 for the 10 µg/mL standard concentration. This intensity was obtained for betulinic acid and ferulic acid using parameters commonly applied for small-molecule analysis on the LC/ESI-/HRMS instrument used here. For ESI+, the parameters were adjusted until the desired peak intensities for betulin and lupeol were also obtained. The scan range was 100-1000 *m/z*, and the resolution was 120,000 (MS^1) or 15,000 (MS^2). For fragmentation, the 5 most intense ions were fragmented in each full-scan, and a stepped 10/40/70 V collision energy was used.

Data analysis

For data analysis, Thermo Xcalibur Qual Browser v. 3.1.66.10 (Thermo Fisher Scientific) was used. Mass tolerance of 5 ppm was used for extracting ion chromatograms, and Gaussian smoothing with 7 points was enabled. For peak area determination, manual integration was used.

Identification

Identification of the compounds was done based on retention time, full-scan (MS^1) and fragmentation spectra (MS^2) matching with the corresponding analytical standard. Betulin and lupeol yielded the highest signal when analysed with ESI+ and were detected as $[M+H]^+$ and $[M-H_2O+H]^+$ ions; however, $[M-H_2O+H]^+$ yielded a higher number of fragment ions and was therefore used for betulin and lupeol identification by MS^2.

Betulin

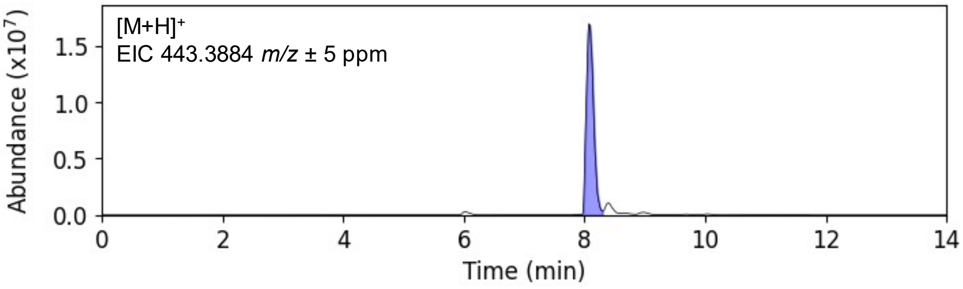

Figure S1. Extracted ion chromatogram of betulin ([M+H]$^+$) in the bark-wood extract (8.45 µg/mL in methanol) measured with ESI+.

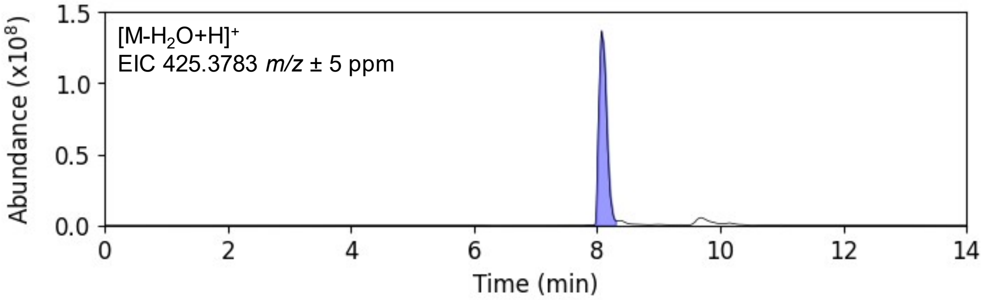

Figure S2. Extracted ion chromatogram of betulin ([M-H_2O+H]$^+$) in the bark-wood extract (8.45 µg/mL in methanol) measured with ESI+.

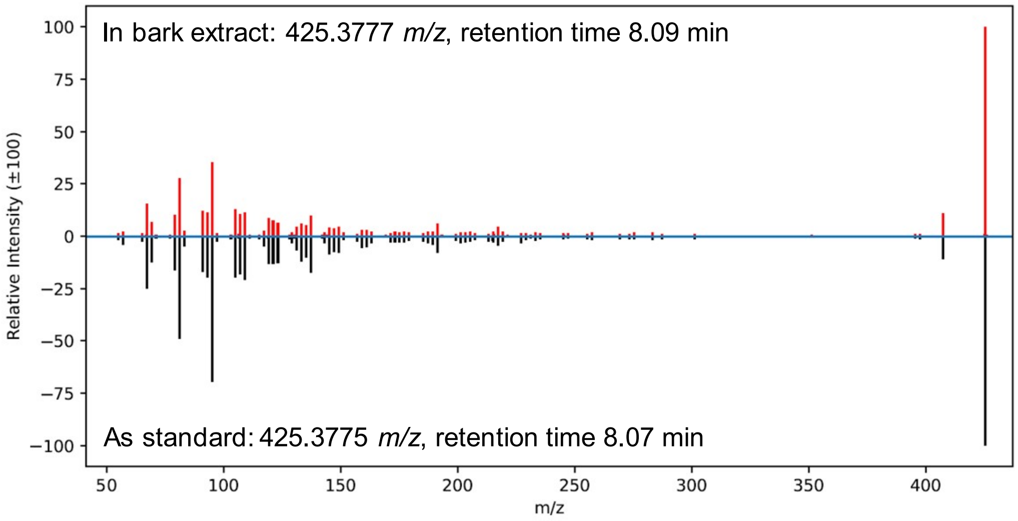

Figure S3. Identification of betulin based on MS^1, MS^2, and retention time using the [M-H_2O+H]$^+$ molecular ion. The mirror plot shows the information for betulin measured in the bark-wood extract (top) and the betulin standard (bottom). The concentration of the bark-wood extract and betulin standard was 15.62 µg/mL and 13.24 µg/mL in methanol, respectively

Lupeol

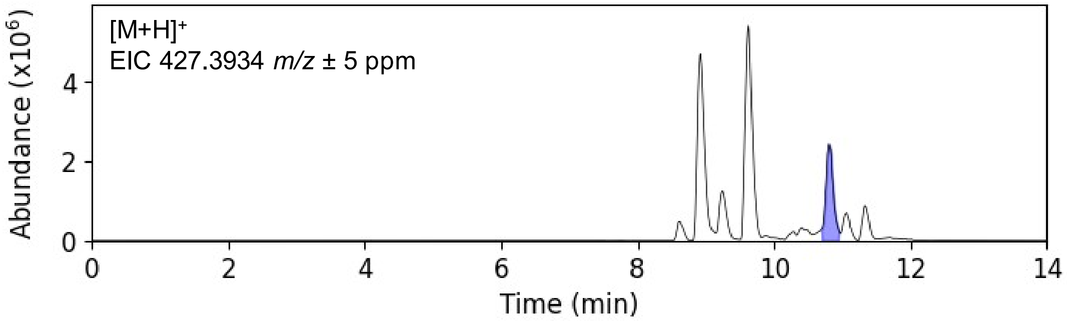

Figure S4. Extracted ion chromatogram of lupeol ([M+H]$^+$) in the bark-wood extract (94.54 µg/mL in methanol) measured with ESI+.

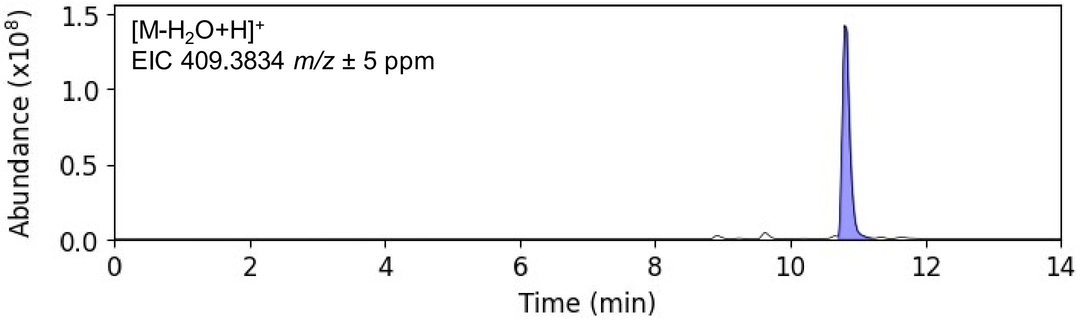

Figure S5. Extracted ion chromatogram of lupeol ([M-H_2O+H]$^+$) in the bark-wood extract (94.54 µg/mL in methanol) measured with ESI+.

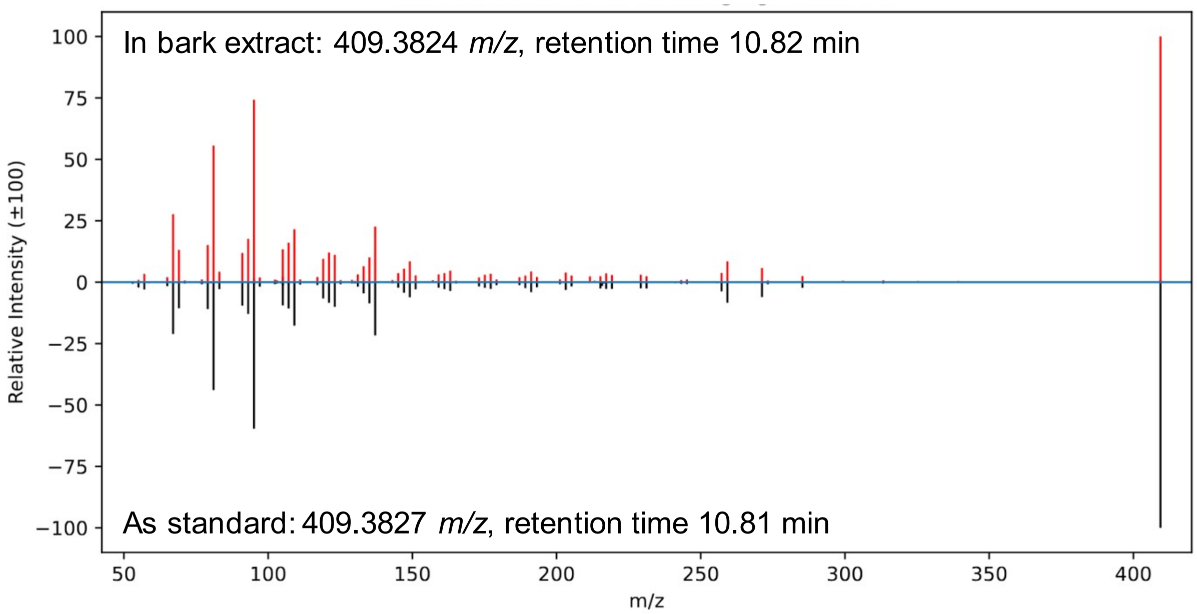

Figure S6. Identification of lupeol based on MS^1, MS^2, and retention time using the [M-H_2O+H]$^+$ molecular ion. The mirror plot shows the information for lupeol measured in the bark-wood extract (top) and the lupeol standard (bottom). The concentration of the bark-wood extract and lupeol standard was 1.06 mg/mL and 10.38 µg/mL in methanol, respectively.

Betulinic acid

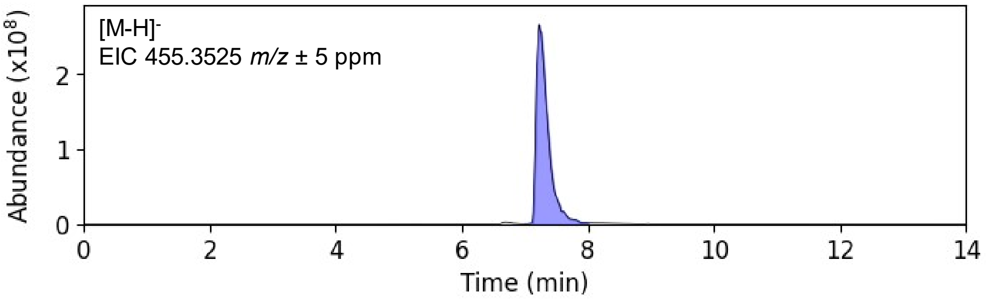

Betulinic acid yielded the highest signal as the $[M-H]^-$ ion, measured by ESI-.

Figure S7. Extracted ion chromatogram of betulinic acid ($[M-H]^-$) in the bark-wood extract (1.06 mg/mL in methanol) measured with ESI-.

For MS^2-based matching, however, the $[M+H]^+$ ion detected in ESI+ yielded the highest

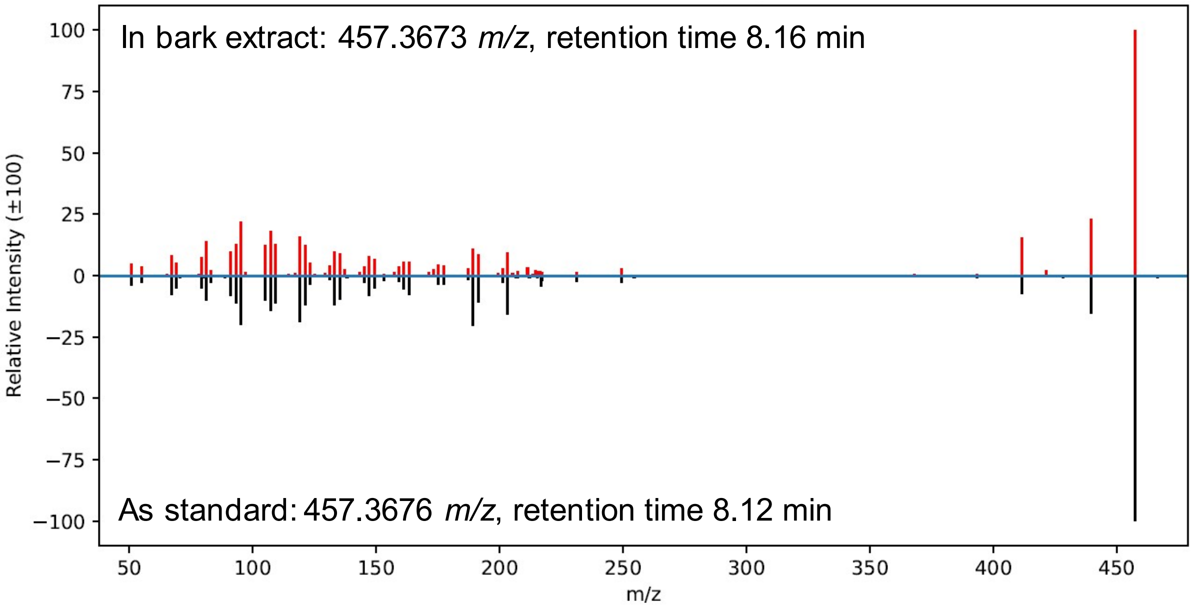

number of fragment ions.

Figure S8. Identification of betulinic acid based on MS^1, MS^2, and retention time using the $[M+H]^+$ molecular ion. The mirror plot shows the information for betulinic acid measured in the bark-wood extract (top) and the betulinic acid standard (bottom). The concentration of the bark-wood extract and betulinic acid standard was 1.06 mg/mL and 13.39 µg/mL in methanol, respectively.

Ferulic acid

Ferulic acid was detected only in ESI- mode, but the signal in the sample was too low to yield MS^2. Thus, the identification of ferulic acid was based on retention time (0.98 min in the bark-wood extract and 0.99 min in the standard) and MS^1 (193.0509 *m/z* in the bark-wood extract and 193.0509 *m/z* in the standard) matching.

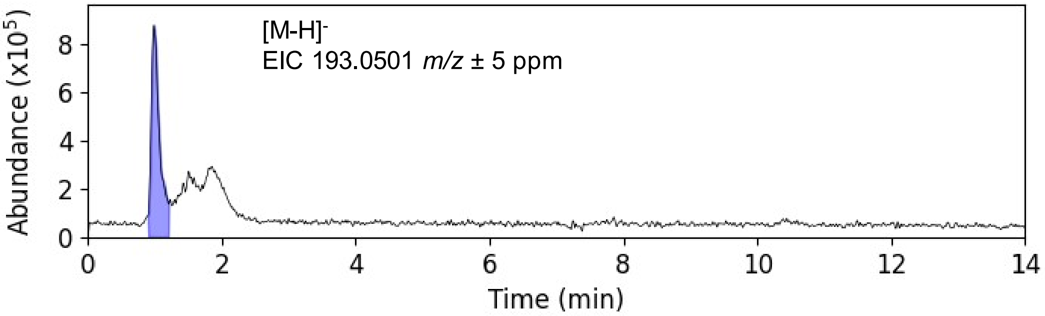

Figure S9. Extracted ion chromatogram of ferulic acid ($[M-H]^-$) in the bark-wood extract (1.06 mg/mL in methanol) measured with ESI-.

Quantification

For quantification, external calibration was used by measuring calibration solutions in methanol with analyte concentrations ranging from 0.0014 to 14.46 µg/mL. Based on these results, calibration graphs were constructed. All calibration solutions and bark-wood extracts were prepared by weighing all components.

The bark-wood extract was evaporated to dryness and redissolved in methanol, yielding a concentration of 1.057 mg/mL (undiluted). From this, different dilutions were made to match the linear range of each analyte: 10× diluted (94.54 µg/mL), 50× diluted (15.62 µg/mL), 100× diluted (8.45 µg/mL), and 1000× diluted (0.75 µg/mL). The concentration of betulin ranged from 2.25 to 13.24 µg/mL in the calibration solutions, lupeol from 2.24 to 14.46 µg/mL, betulinic acid from 2.67 to 13.39 µg/mL, and ferulic acid from 0.0014 to 0.070 µg/mL.

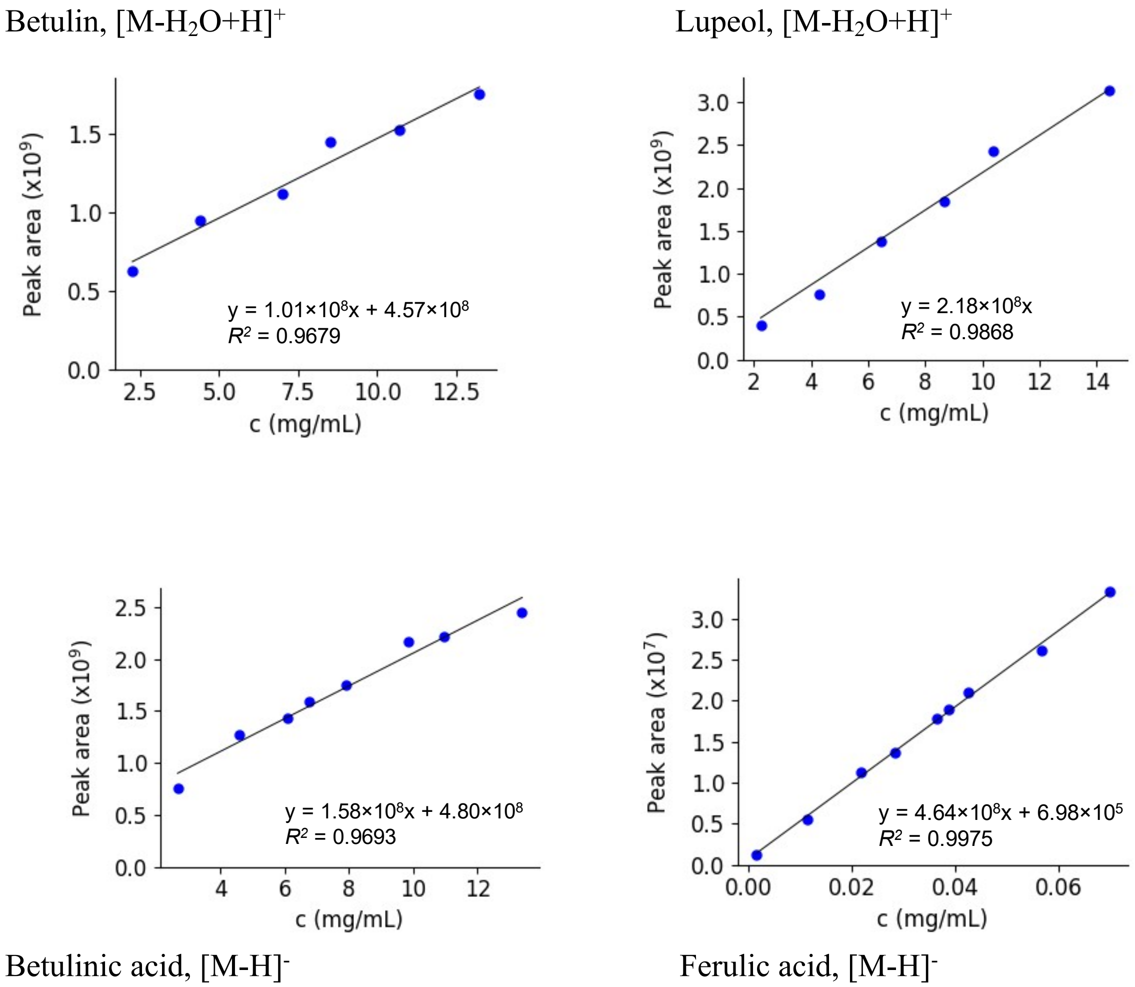

Figure S10. Calibration graphs for betulin, lupeol, betulinic acid, and ferulic acid

For betulin, the $[M\text{-}H_2O+H]^+$ peak area was within the linear range of the calibration graph for the 50× and 100× dilutions. **Betulin** concentration was calculated to be **89.7 g per 100 g of dried bark-wood extract** (i.e., 89.7%; n=2). For lupeol, the $[M\text{-}H_2O+H]^+$ peak area was within the linear range of the calibration graph for the 10× dilution. **Lupeol concentration** was calculated to be **5.4 g per 100 g of dried bark-wood extract** (i.e., 5.4%; n=1). For betulinic and ferulic acid, the $[M\text{-}H]^-$ peak area was within the linear range of the calibration graph for the undiluted sample. **Betulinic acid** concentration was calculated to be **1.8 g per 100 g of dried bark-wood extract** (i.e., 1.8%; n=1). **Ferulic acid** concentration was calculated to be **0.0016 g per 100 g of dried bark-wood extract** (i.e., 0.0016%; n=1).

S.4. Suberin

Extractive-free biomass sample (350 mg) was refluxed with 3% MeONa in MeOH (25 mL) for 2 hours. After the completion, the mixture was centrifugated and residue was washed with MeOH and water until neutral pH. Solid residue was separated and dried overnight. The

remaining solution was acidified with H_2SO_4 until pH 2-3 and extracted with DCM (3*10 mL). The organic fraction was dried, filtered and concentrated to yield suberin.

S.5. Reductive catalytic fractionation (RCF)

The extracted biomass was then subjected to the RCF. In a typical experiment 200 mg of biomass together with 20 mg (10 wt%) of 5% Pd/C is loaded in a stainless steel Svagelok reactor. 8 ml of MeOH:H_2O (7:3) containing 1,1 g/L pTSA together with 80 mg of HCOOH is added to the reactor. Finally, a small stirring magnet is placed and the reactor is tightly closed and submerged in an oil bath heated to 200 ^{0}C. The reaction is heated for 4 h. After 4 h the reactor is cooled down to room temperature under cold tap water. The reaction mixture is filtered, washed with DI water and methanol mixture, the solid residue is then dried in the oven at 60 ^{0}C overnight and subjected to further analysis. The filtrate is collected, solvent is removed under reduced pressure, the resulting oil is weighed and diluted with EtOAc to the desired concentration. 1 mL of solution together with internal standard (tetracosane) are added to the GC vials and analysed by GC-MS/FID.

GC-MS and GC-FID analyses were conducted by a QP2020 system (SHIMADZU, Japan) equipped with two parallel HP-5MS columns (30 m × 0.25 mm × 0.25 μm). GC-MS/FID condition: injector was opened at 250 ˚C. The column temperature program: 60 C (2 min), from 60 ^{o}C to 270 ^{o}C (15 ^{o}C·min^{-1}), 270 ^{o}C (5 min), from 270 ^{o}C to 300 ^{o}C (5 ^{o}C·min^{-1}), 300 ^{o}C (10 min). Mobile phase: helium at a rate 1.46 ml·min^{-1}.

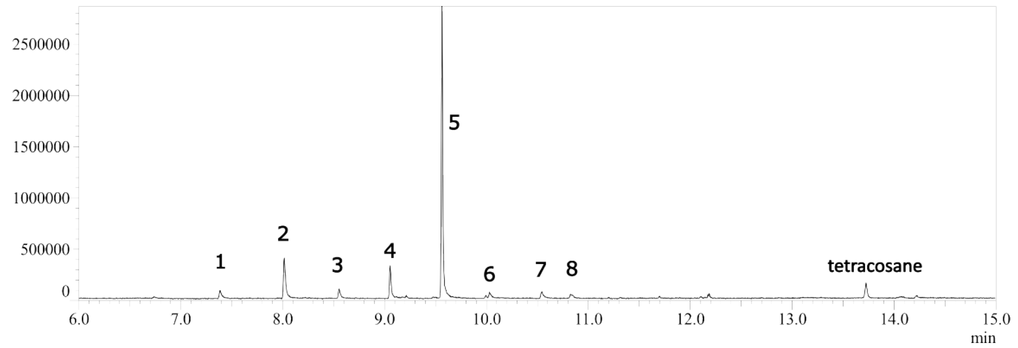

Figure S11. GC-FID chromatogram of monophenolic compounds in RCF product mixture.

Lignin monomer yield was calculated following *Nature protocol*.[1] Percentage of each monophenolic compounds was calculated based on tetracosane standard addition by comparing peak area in GC-FID. Effective carbon number (ECN) factors were applied to the calculation

and contribution effect was shown in Table S1. Each monophenolic compound was calculated by following [Eq 1-3]. The results are shown in Figure S12.

$$N_{monomer} = \frac{A_{monomer}}{A_{tetracosane}} \times N_{dodecane} \times \frac{ECN_{tetracosane}}{ECN_{monomer}} \quad \text{[Eq.1]}$$

$$m_{monomer} = N_{monomer} \times MW_{monomer} \quad \text{[Eq.2]}$$

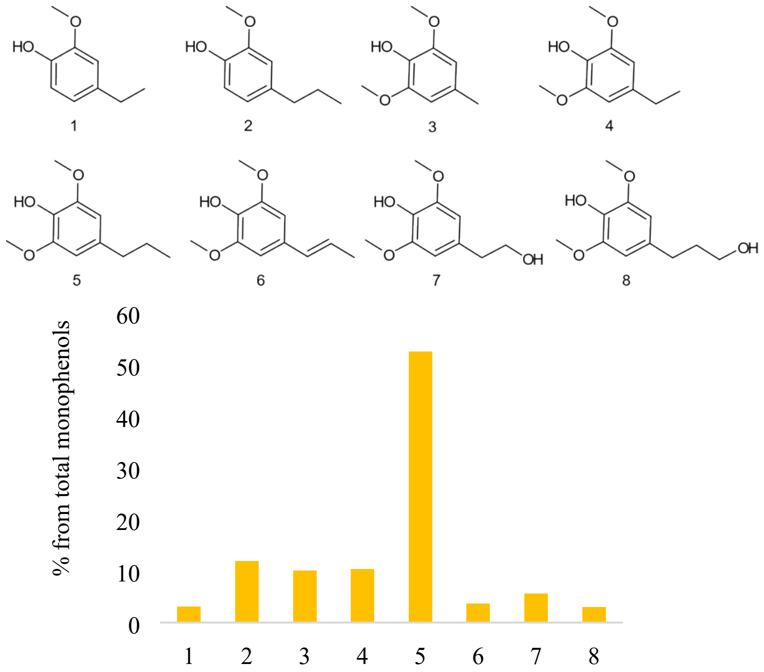

Figure 12. Distribution of monomers in RCF product mixture.

$$Yield_{monomer} = \frac{m_{monomer}}{m_{biomass} \times \text{klason lignin content (AIL)}} \times 100\% \quad \text{[Eq.3]}$$

Whereas; N (moles), A (integrated are of peak), ECN (effective carbon number), MW (molecular weight), and m (mass).

Table S1. Contribution effect of effective carbon number

Atom/Group	ECN contribution
Oxygen/Phenol	-1
Carbon/Aromatic	1
Carbon/Aliphatic	1

S.6. Organocatalysis of the remaining solid residue

The dry solid residue remaining after RCF is then subjected to the basic catalysis in the presence of Et_3N to depolymerize suberin. In a typical experiment 300 mg of solid residue

together with 4,5 ml (15 mL/g) of solvent (MeOH:H2O:NEt_3 46:47:7) and a stirring magnet are placed into a stainless steel Swagelok reactor. The reactor is tightly sealed and submerged into preheated to 220 ºC oil bath for 2 h. After completion of the reaction the mixture is filtered through filter paper, washed with methanol and water, the filtrate is acidified to pH 2-3, extracted with DCM (3*10 mL) and concentrated under the reduced pressure to yield suberin. The solid residue is dried in the oven at 60 ºC overnight and weighed. The resulting solid is then dissolved in THF to the approximate concentration of 1 mg/mL and analysed by GPC to detect the distribution of the molecular weight in the product mixture.

GPC analysis was performed on a Prominence-i, LC-2030C system (SHIMADZU, Japan) equipped with a UV detector a 280 nm.

S.7. Monomeric composition of suberin. LC-HRMS analysis of the fatty acid mixture obtained from suberin hydrolysis

To detect the monomeric composition of suberin, 100 mg of suberin was refluxed for 1h in 5 ml of 3% KOH in MeOH. After the completion of the reaction, the product mixture was acidified with 1M HCL to pH 2-3 and extracted with DCM (3*10 mL). Organic fractions were collected, solvent was evaporated and the resulting product mixture was analysed by LC-HRMS.

Experimental parameters

The LC-HRMS experiments were performed on a Vanquish UHPLC system hyphenated to an Orbitrap Exploris 480 HRMS (both from Thermo Fisher Scientific). For LC separation, the Kinetex F5 Core-Shell column was used, with the following conditions: flow rate 0.30 mL/min, injection volume 5 μL, and oven temperature 40 °C. ESI- mode was used, and the mobile phases were water (A) and methanol (B), both with 2 mM ammonium acetate. Gradient elution started at 1% B, increased to 99% B in 30 min, held for 1 min, decreased back to 1% B in 0.5 min, and equilibrated for 1.5 min, with a total run time of 33 min. For the HRMS, the following parameters were used: ESI- mode, spray voltage 3 kV, ion transfer tube temperature 275 °C, vaporiser temperature 275 °C, aux gas flow rate 5 AU, sheath gas flow rate 30 AU, and RF lens 50%. The scan range was 75-1000 *m/z*, and the resolution was 120,000 (MS^1) or 15,000 (MS^2). Dynamic exclusion in automatic mode and apex detection, with 30% of the desired apex window, were applied. The 5 most intense ions were fragmented in each full-scan, and lock mass correction was applied at the start of each run (0-1 min). The sample was analysed four times by changing only the MS^2 collision energy: 20V, 35V, 50V, and a stepped

10/40/70 V. Also, a blank sample (pure methanol) was measured under the same parameters, with a 35V collision energy.

Data processing

The raw measurement data were converted to mzML files using MSConvertGUI[2] from ProteoWizard (proteowizard.sourceforge.io). The mzML files were processed with MZMine[3] (v. 4.7.8) from mzio GmbH (Bremen, Germany). Blank subtraction was done by only keeping compounds that had at least 3× higher abundance in the sample than in the blank and had a minimum peak height of 1×10^7. The detected compounds were then exported for structural annotation into SIRUS[4](v. 6.1.0), where 5 ppm was selected for MS^2 mass accuracy. The MS^2 data obtained with different collision energies for each compound were automatically merged in SIRIUS. The structures are proposed by comparing molecular fingerprints predicted from experimental MS^2 data with fingerprints of candidate structures retrieved from databases. All databases available in SIRIUS were enabled: PubChem, Biocyc, Blood Exposome, CHEBI, COCONUT, DSSTox, FooDB, GNPS, HMDB, HSDB, KEGG, KNapSAcK, LOTUS, LipidMaps, Maconda, MeSH, MiMeDB, Plantcyc, PubMed, SuperNatural, TeroMOL, YMDB. This enabled proposing the overall structure of the compounds (e.g., dihydroxyhexadecanoic acid); however, for some compounds, determining the exact positional isomers can be difficult (e.g., whether it's 10,16-dihydroxyhexadecanoic acid or 8,12-dihydroxyhexadecanoic acid). Reference spectra for dihydroxy(n)dioic acids and epoxy(n)oic acids were also searched in MassBank and MONA, but none suitable for comparison with the experimental data obtained in this work were found.

Tentative identification

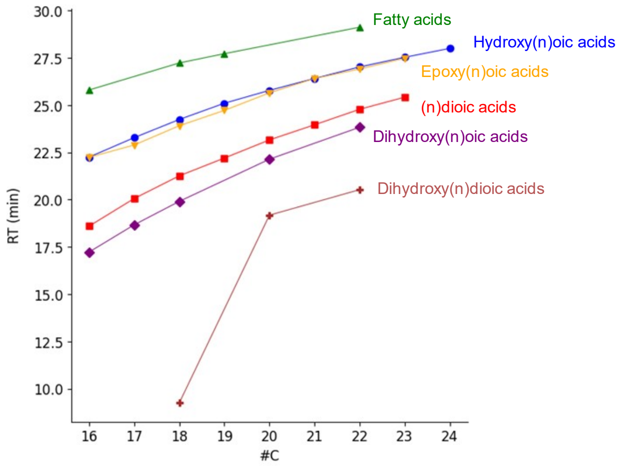

Figure S14. The Retention time (RT) plotted against carbon number (#C) for fatty acid derivative classes containing at least three compounds to evaluate whether compounds differing only in chain length follow a consistent elution trend.

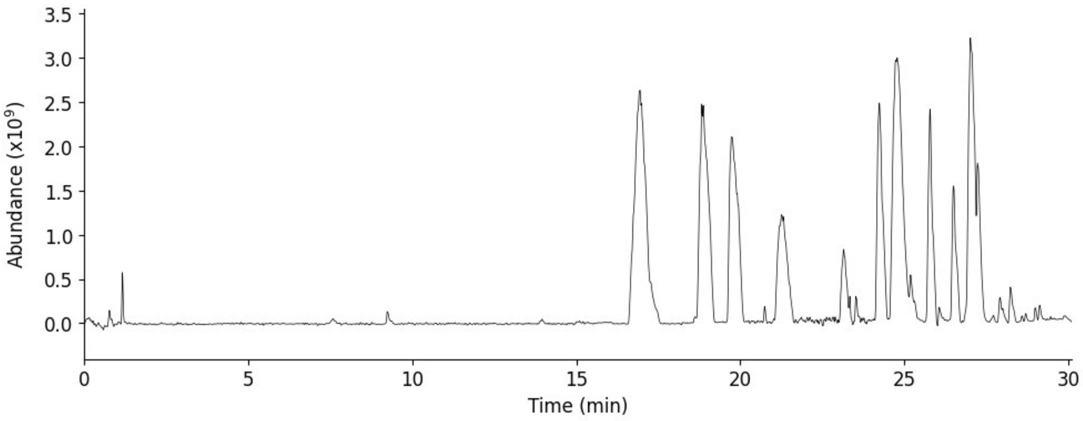

Figure S13. Chromatogram of the hydrolysed fatty acid mixture from suberin. Base peak chromatogram with the blank chromatogram subtracted.

The elution order of fatty acid derivatives within the same group, differing only in carbon number (#C), should follow a consistent trend. Thus, the retention time (RT) was plotted against #C for each compound class containing at least 3 compounds to provide additional confirmation of the class assignment of the compounds (Figure S14). Most compounds follow the expected trend; however, the C18 dihydroxy(n)dioic acid deviates from this pattern and appears as an outlier. This may indicate that additional interactions influence its retention behaviour, or that the compound differs from the others in more than just carbon number (e.g., branching or the position of the OH group).

Table S2. Extracted ion chromatograms (EIC) of the fatty acid derivatives and the proposed identifications. In each chromatogram, the purple-shaded peak is considered the match. Some chromatograms also show other non-shaded peaks – these correspond to isomers having the same molecular formula. Δ*m/z* – mass error between measured and calculated *m/z* of the molecular ion in ppm. RT – retention time. The formula represents the assigned molecular formula, noted as M when describing the detected ion ($[M-H]^-$).

Fatty acids	
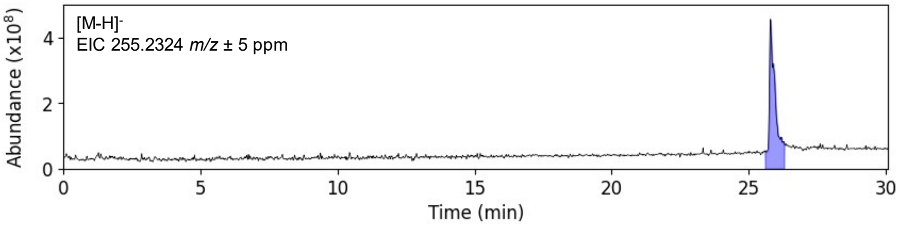	$C_{16}H_{34}O_2$<br>Hexadecanoic acid<br>RT = 25.80 min<br>$\Delta m/z$ = 1.570 ppm
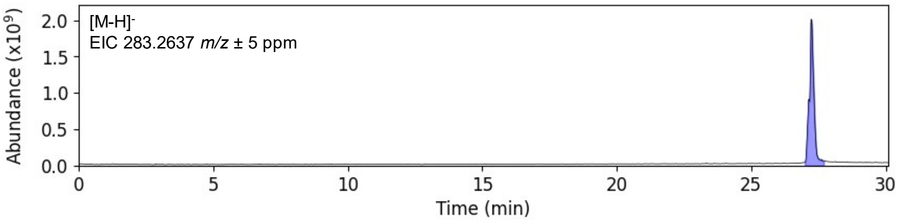	$C_{18}H_{36}O_2$<br>Octadecanoic acid<br>RT = 27.23 min<br>$\Delta m/z$ = -0.378 ppm
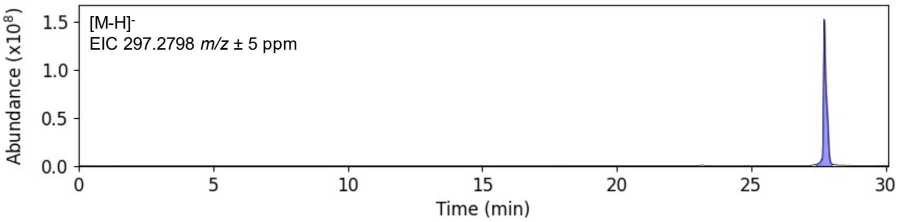	$C_{19}H_{38}O_2$<br>Nonadecylic acid<br>RT = 27.72 min<br>$\Delta m/z$ = -0.978 ppm
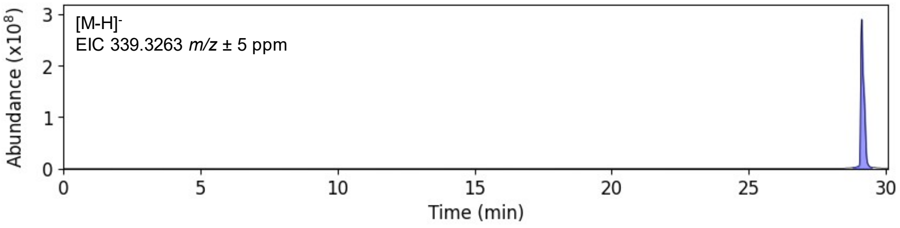	$C_{22}H_{44}O_2$<br>Docosanoic acid<br>RT = 29.12 min<br>$\Delta m/z$ = -1.704 ppm
(n)dioic acids	
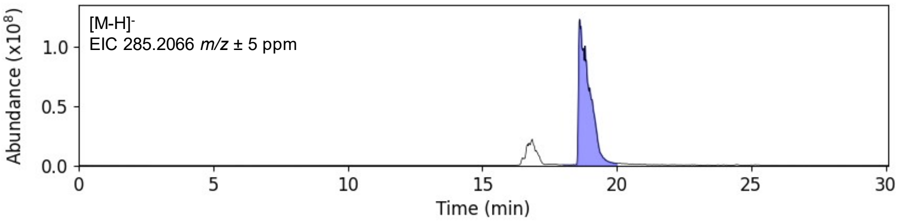	$C_{16}H_{30}O_4$<br>Hexadecanedioic acid<br>RT = 18.61 min<br>$\Delta m/z$ = -0.701 ppm
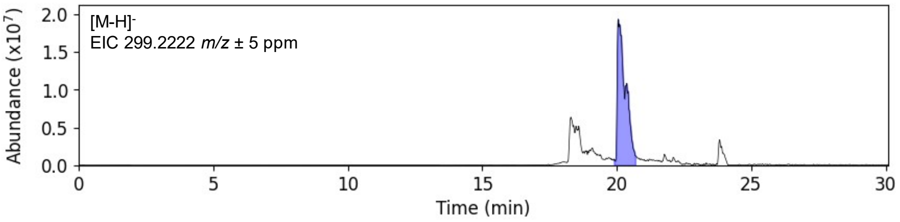	$C_{17}H_{32}O_4$<br>Heptadecadioic acid<br>RT = 20.06<br>$\Delta m/z$ = -0.824 ppm

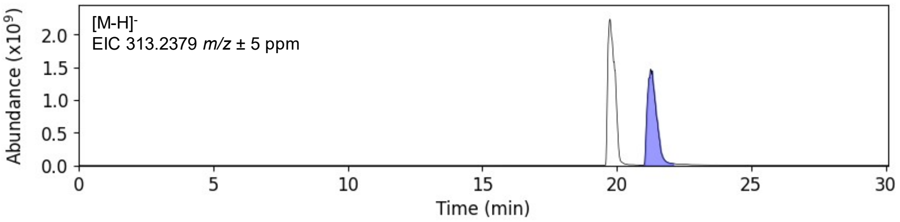

$C_{18}H_{34}O_4$
Octadecanedioic acid
RT = 21.26 min
Δ*m/z* = -0.550 ppm

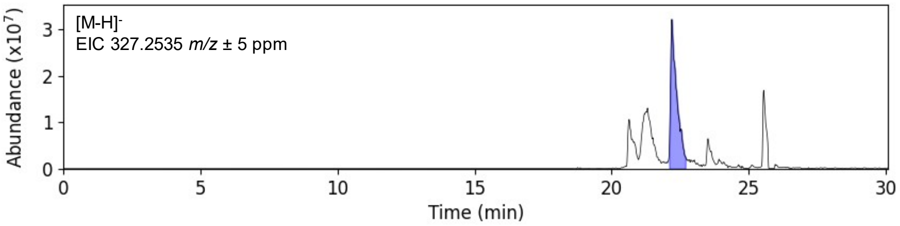

$C_{19}H_{36}O_4$
Nonadecanedioic acid
RT = 22.20 min
Δ*m/z* = -1.279 ppm

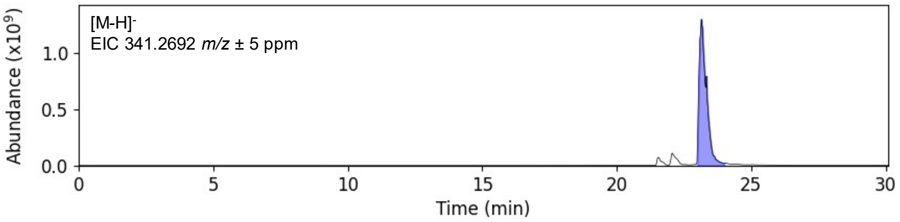

$C_{20}H_{38}O_4$
Eicosanedioic acid
RT = 23.16 min
Δ*m/z* = 0.397 ppm

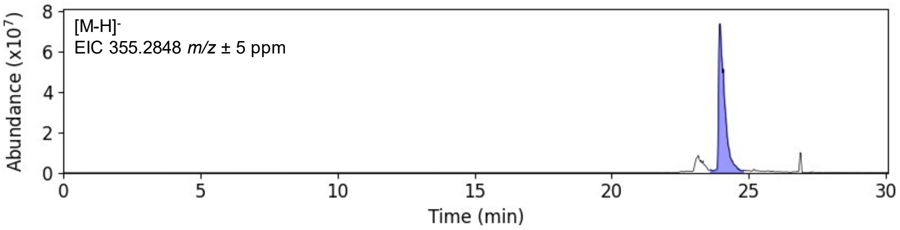

$C_{21}H_{40}O_4$
Heneicosanedioic acid
RT = 23.96 min
Δ*m/z* = -1.562 ppm

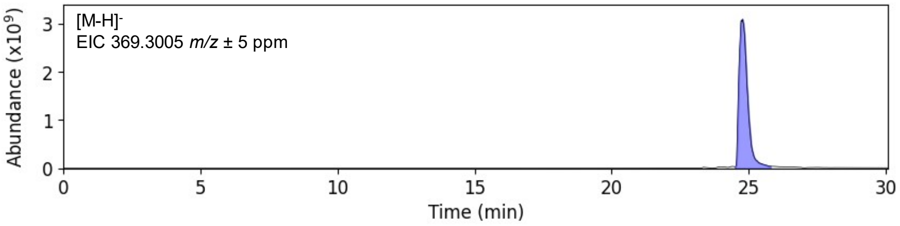

$C_{22}H_{42}O_4$
Docosanedioic acid
RT = 24.78 min
Δ*m/z* = -1.945 ppm

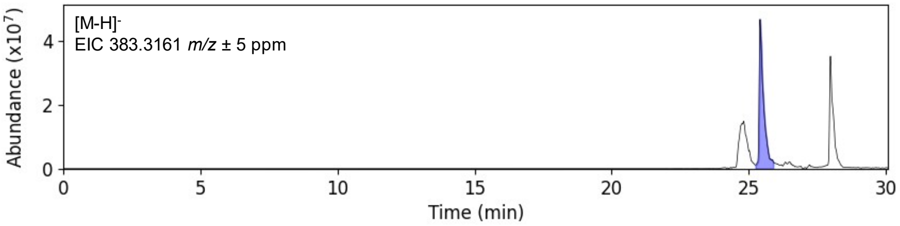

$C_{23}H_{44}O_4$
Tricosanedioic acid
RT = 25.42 min
Δ*m/z* = -1.633 ppm

Hydroxy(n)oic acids

Based on prior knowledge of fatty acid composition in suberin and the consistent RT vs #C trendline, we propose that in the compounds assigned to the shaded peaks, the hydroxyl group is in the ω-position.

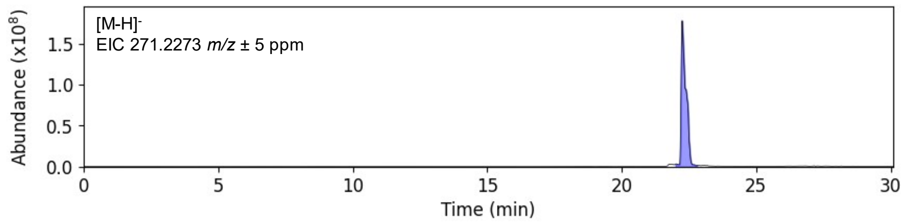

$C_{16}H_{32}O_3$
Hydroxy-hexadecanoic acid
RT = 22.25 min
$\Delta m/z$ = -0.109 ppm

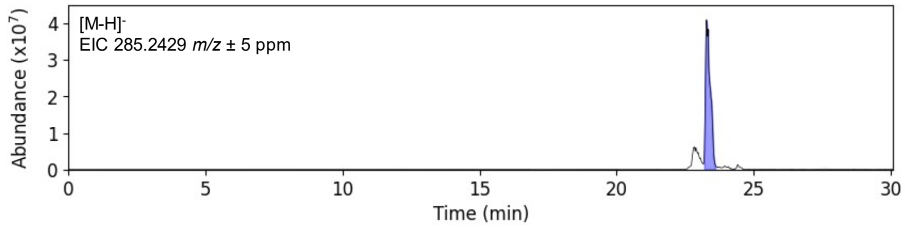

$C_{17}H_{34}O_3$
Hydroxy-heptadecanoic acid
RT = 23.28 min
$\Delta m/z$ = -1.708 ppm

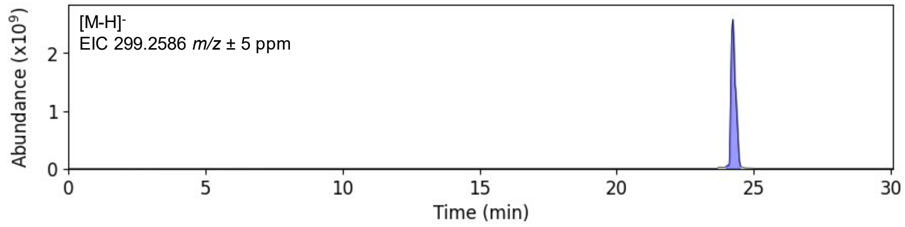

$C_{18}H_{36}O_3$
Hydroxy-octadecanoic acid
RT = 24.24 min
$\Delta m/z$ = -0.156 ppm

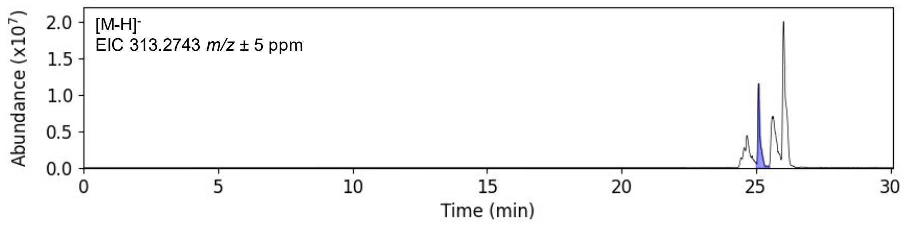

$C_{19}H_{38}O_3$
Hydroxy-nonadecanoic acid
RT = 25.10 min
$\Delta m/z$ = -1.440 ppm

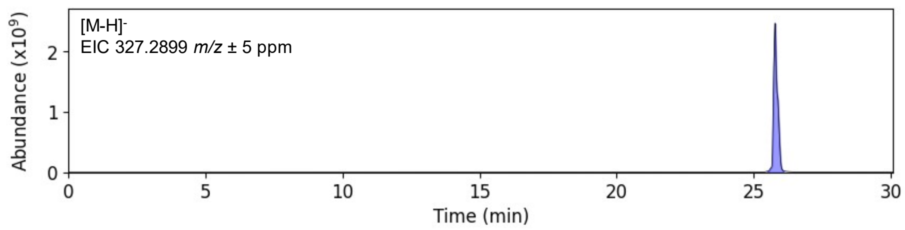

$C_{20}H_{40}O_3$
Hydroxy-eicosanoic acid
RT = 25.78 min
$\Delta m/z$ = -0.787 ppm

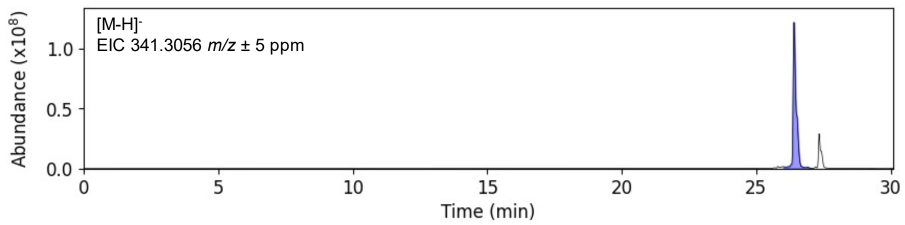

$C_{21}H_{42}O_3$
Hydroxy-heneicosanoic acid
RT = 26.41 min
$\Delta m/z$ = -1.625 ppm

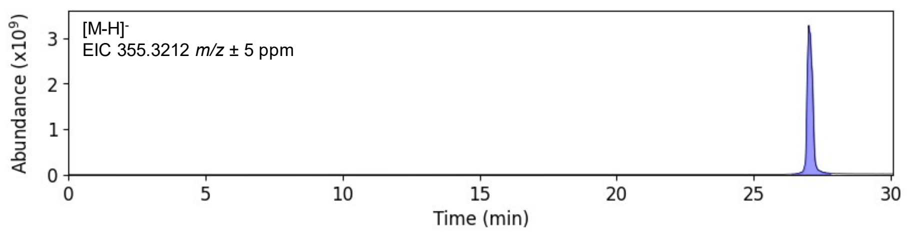

$C_{22}H_{44}O_3$
Hydroxy-docosanoic acid
RT = 27.02 min
Δ*m/z* = -1.876 ppm

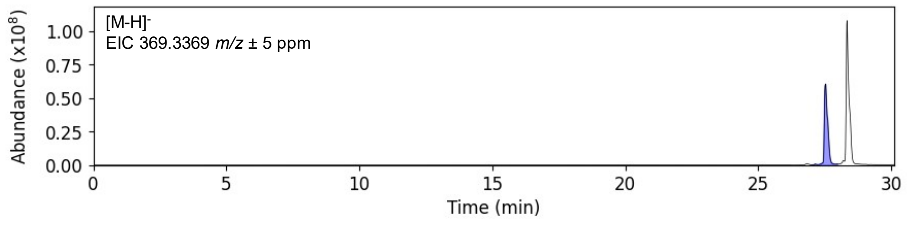

$C_{23}H_{46}O_3$
Hydroxy-tricosanoic acid
RT = 27.53 min
Δ*m/z* = -0.467 ppm

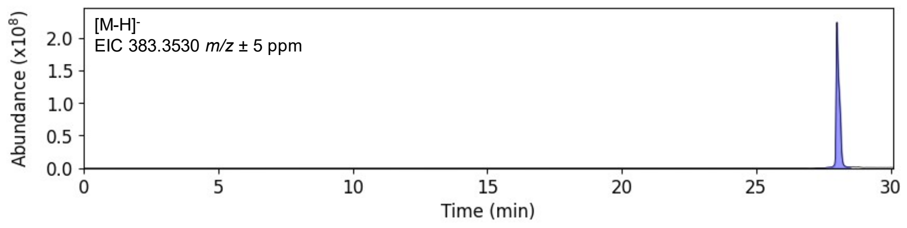

$C_{24}H_{48}O_3$
Hydroxy-tetracosanoic acid
RT = 28.00 min
Δ*m/z* = -0.561 ppm

<u>(n)enedioic acids</u>

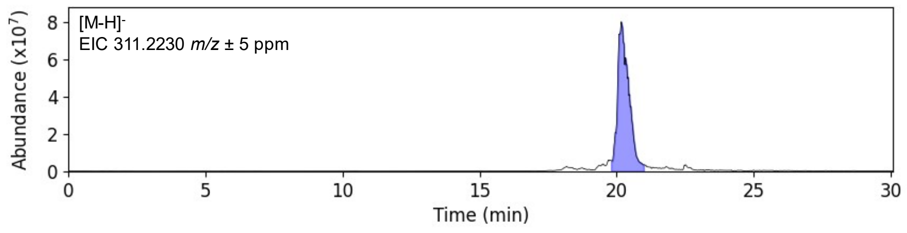

$C_{18}H_{32}O_4$
Octadecenedioic acid
RT = 20.17 min
Δ*m/z* = -0.767 ppm

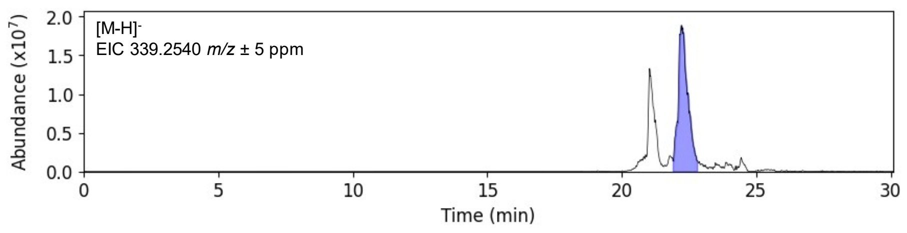

$C_{20}H_{36}O_4$
Eicosenedioic acid
RT = 23.16 min
Δ*m/z* = -0.880 ppm

<u>Epoxy(n)oic acids</u>

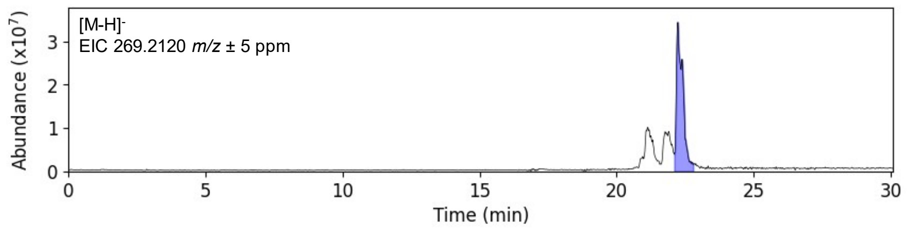

$C_{16}H_{30}O_3$
Epoxyhexadecanoic acid
RT = 22.24 min
Δ*m/z* = -0.922 ppm

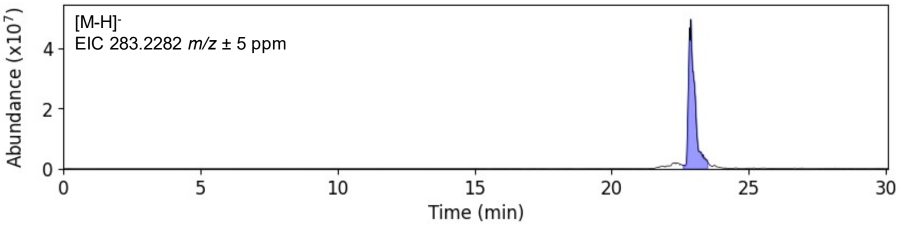	$C_{17}H_{32}O_3$<br>Epoxyheptadecanoic acid<br>RT = 22.89 min<br>Δ*m/z* = 1.205 ppm
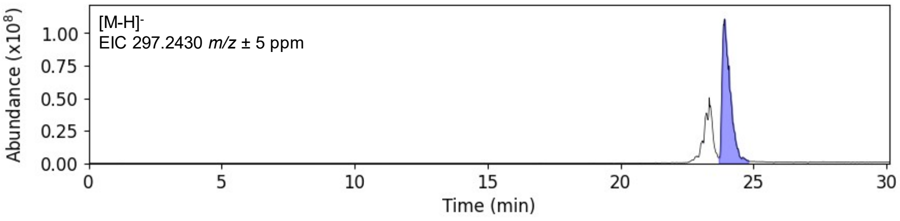	$C_{18}H_{34}O_3$<br>Epoxyoctadecanoic acid<br>RT = 23.91 min<br>Δ*m/z* = -0.626 ppm
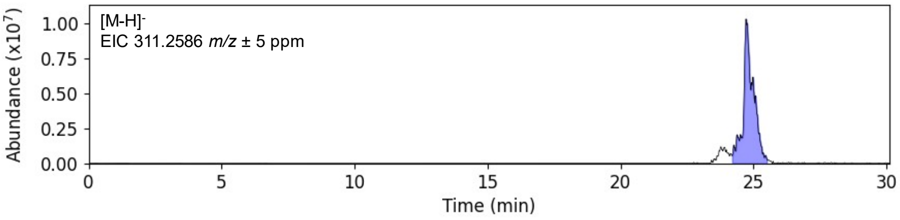	$C_{19}H_{36}O_3$<br>Epoxynonadecanoic acid<br>RT = 24.72 min<br>Δ*m/z* = 0.496 ppm
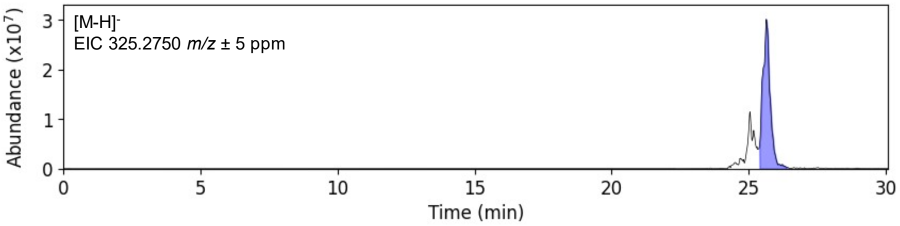	$C_{20}H_{38}O_3$<br>Epoxyeicosanoic acid<br>RT = 25.65 min<br>Δ*m/z* = -1.312 ppm
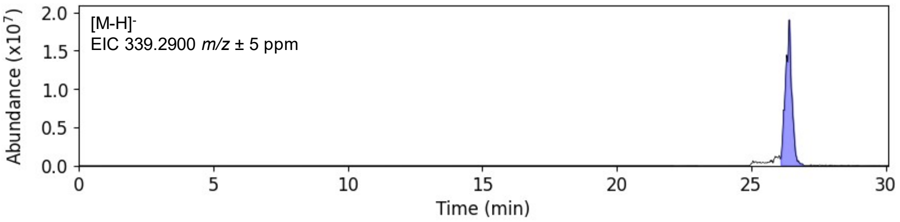	$C_{21}H_{40}O_3$<br>Epoxyheneicosanoic acid<br>RT = 26.41 min<br>Δ*m/z* = -0.146 ppm
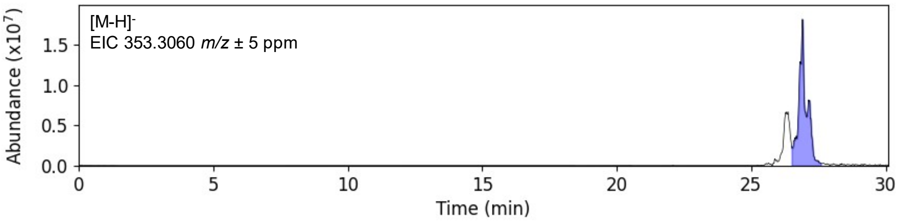	$C_{22}H_{42}O_3$<br>Epoxydocosanoic acid<br>RT = 26.91 min<br>Δ*m/z* = -0.956 ppm

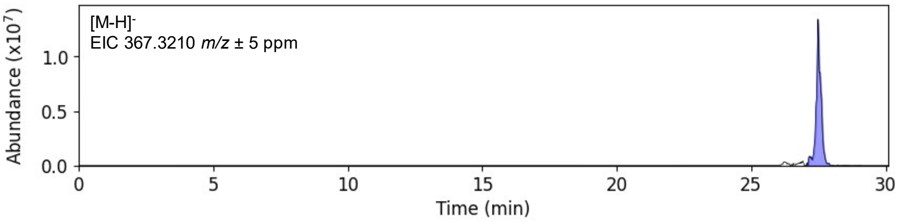

$C_{23}H_{44}O_3$
Epoxytricosanoic acid
RT = 27.48 min
Δ*m/z* = 1.075 ppm

Dihydroxy(n)dioic acids

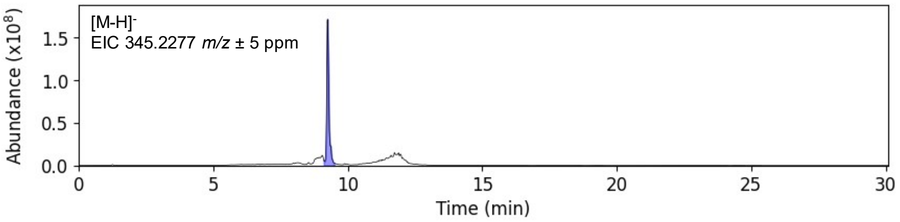

$C_{18}H_{34}O_6$
Dihydroxy-octadecanedioic acid
RT = 9.25 min
Δ*m/z* = 0.166 ppm

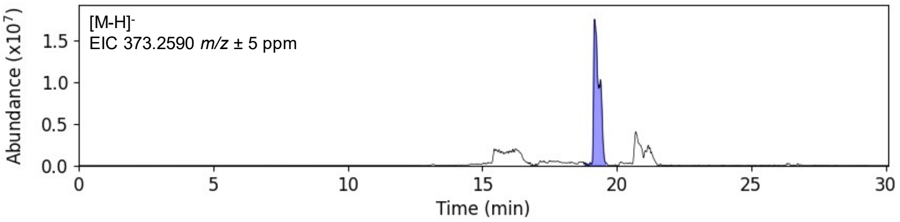

$C_{20}H_{38}O_6$
Dihydroxy-eicosanedioic acid
RT = 19.18 min
Δ*m/z* = -0.607 ppm

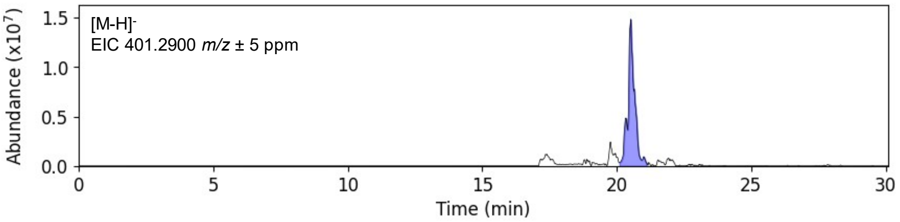

$C_{22}H_{42}O_6$
Dihydroxy-docosanedioic acid
RT = 20.53 min
Δ*m/z* = 0.281 ppm

Dihydroxy(n)oic acids

Based on prior knowledge of fatty acid composition in suberin and the consistent RT vs #C trendline, we propose that in the compounds assigned to the shaded peaks, one of the hydroxyl groups is in the ω-position.

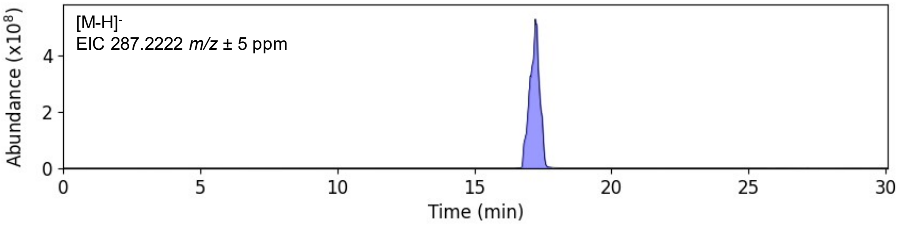

$C_{16}H_{32}O_4$
Dihydroxy-hexadecanoic acid
RT = 17.23 min
Δ*m/z* = -0.397 ppm

<table>
<tr><td>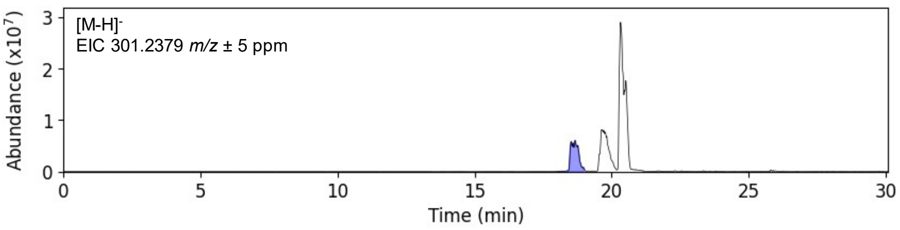
</td><td>$C_{17}H_{34}O_4$
Dihydroxy-heptadecanoic acid
RT = 18.67 min
$\Delta m/z$ = 0.400 ppm</td></tr>
<tr><td>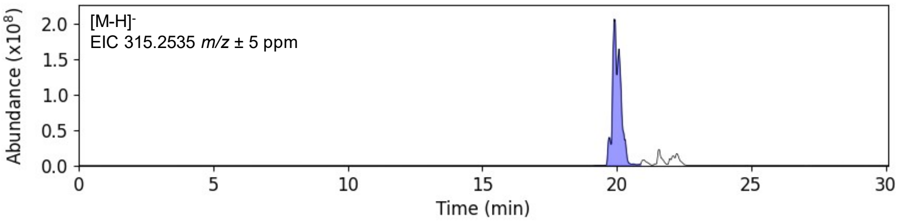
</td><td>$C_{18}H_{36}O_4$
Dihydroxy-octadecanoic acid
RT = 19.91 min
$\Delta m/z$ = 0.873 ppm</td></tr>
<tr><td>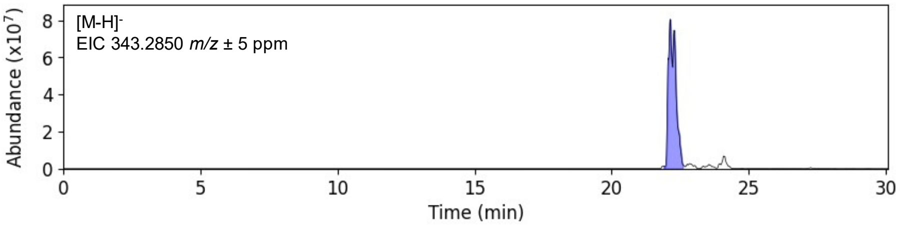
</td><td>$C_{20}H_{40}O_4$
Dihydroxy-eicosanoic acid
RT = 22.14 min
$\Delta m/z$ = -0.912 ppm</td></tr>
<tr><td>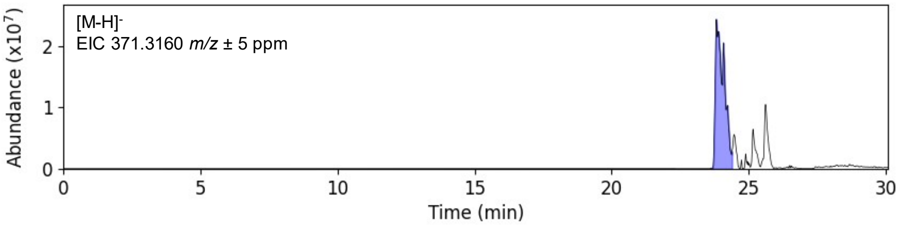
</td><td>$C_{22}H_{44}O_4$
Dihydroxy-docosanoic acid
RT = 23.83 min
$\Delta m/z$ = 0.148 ppm</td></tr>
<tr><td colspan="2"><u>Dihydroxy(n)enoic acids</u>
Based on prior knowledge of fatty acid composition in suberin, we propose that in the compound assigned to the shaded peak, one of the hydroxyl groups is in the ω-position.</td></tr>
<tr><td>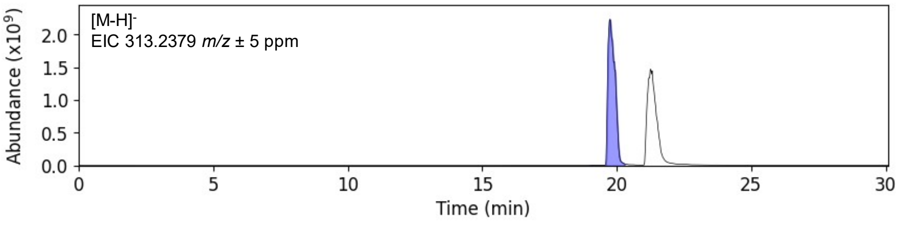
</td><td>$C_{18}H_{34}O_4$
Dihydroxy-octadecenoic acid
RT = 19.75 min
$\Delta m/z$ = -0.652 ppm</td></tr>
<tr><td colspan="2"><u>Trihydroxy(n)oic acids</u>
Based on prior knowledge of fatty acid composition in suberin, we propose that in the compounds assigned to the shaded peaks, one of the hydroxyl groups is in the ω-position.</td></tr>
</table>

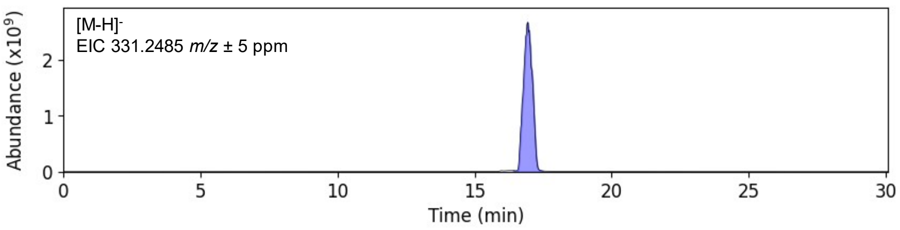

$C_{18}H_{36}O_5$
Trihydroxy-octadecanoic acid
RT = 16.94 min
Δ*m/z* = -0.627 ppm

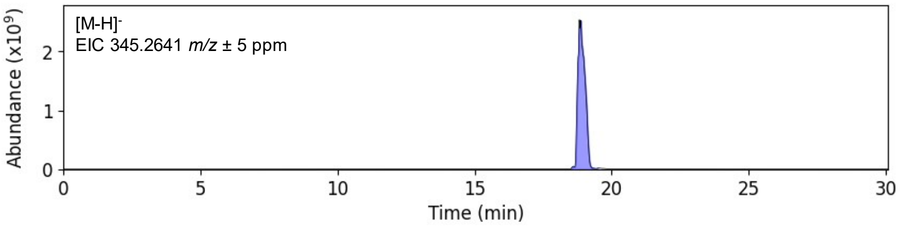

$C_{19}H_{38}O_5$
Trihydroxy-nonadecanoic acid
RT = 18.82 min
Δ*m/z* = -1.149 ppm

Trihydroxy(n)enoic acids

Based on prior knowledge of fatty acid composition in suberin, we propose that in the compounds assigned to the shaded peaks, one of the hydroxyl groups is in the ω-position.

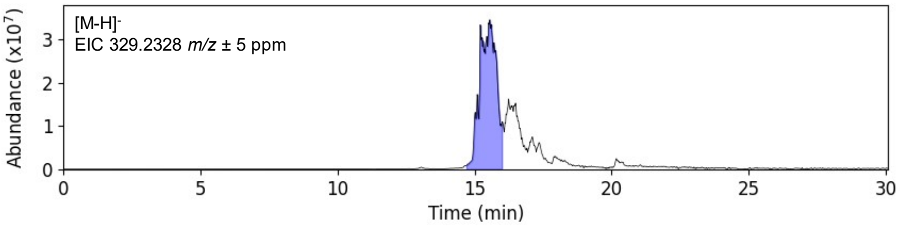

$C_{18}H_{34}O_5$
Trihydroxy-octadecenoic acid
RT = 15.56 min
Δ*m/z* = -0.931 ppm

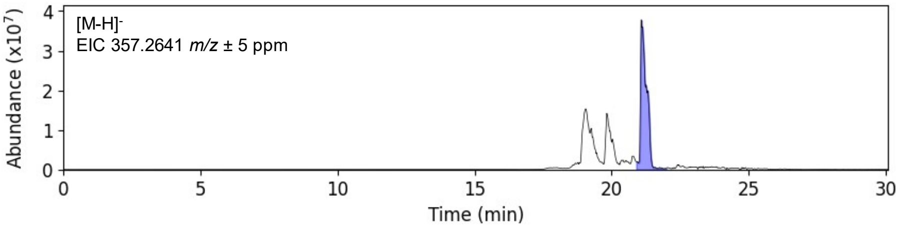

$C_{20}H_{38}O_5$
Trihydroxy-eicosenoic acid
RT = 21.08 min
Δ*m/z* = 0.127 ppm

S.8. Determination of carbohydrates and acid insoluble lignin (Klason method)

Biomass is dried overnight in the oven at 60 ºC. In a typical experiment, a 300 mg sample of biomass is placed in a 100 mL bottle together with 3 mL (4,92 g) of 72% H_2SO_4 and a stirring magnet and tightly sealed. The mixture is stirred at 30 ºC in a water bath for 60 min. After completion, the reaction mixture is diluted with DI water (84 mL) to the concentration of 4%. The diluted mixture is then stirred at 121 ºC for another 60 min. Upon completion, the reaction mixture is cooled down to rt, filtered through filter paper, the filtrate is collected and neutralized using BaCO3 and sample is taken for the HPLC analysis. The remaining solid is washed with DI water until neutral pH and dried in the oven at 60 ºC overnight to yield acid insoluble lignin (AIL).

Sugar analysis was conducted using an Agilent 1200 Series HPLC system equipped with Biorad Aminex HPX-87P column (300 mm × 7.8 mm) coupled with refractive index detector.

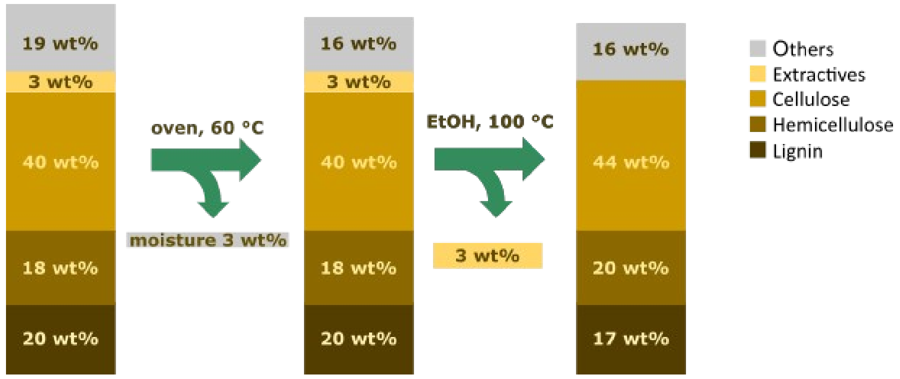

Figure S15. Compositional analysis of birch wood before and after extraction

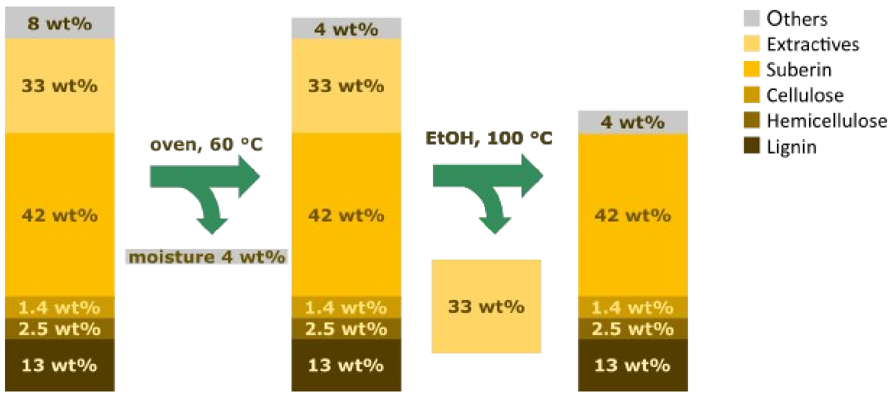

Figure S16. Compositional analysis of birch bark before and after extraction.

S. 9. Control experiment. Thioacidolysis of the solid residue after Et_3N of the biomass (organocatalysis-first).

Thioacidolysis reagent is prepared as follows: 2.5 ml of EtSH was mixed with 0.7 mL of BF_3*Et_2O and diluted with dioxane to 25 mL. In a typical experiment, 40 mg of the biomass (solid residue after organocatalysis) together with 4 mL of the thioacidolysis reagentare added to a microwave vial. A stirring magnet is placed in the same vial and the vial is sealed with a cap and placed in a pre-heated to 100 ºC oil bath for 4 h. After the completion, the reaction mixture is cooled down. A saturated solution of $NaHCO_3$ is added to the reaction mixture, followed by 1M HCl until pH 1-3. The mixture is extracted with DCM, water and brine, dried over $MgSO_4$. The organic phase is filtered, the solvent is removed under reduced pressure and the crude is dissolved in 5 mL of EtOAc. 0.5 of the dissolved crude and o.1 mL of tetracosane solution in EtOAc as internal standard are transferred to a 5 mL glass vial. The solvent was evaporated and 0.1 mL of BSTFA with 0.1 mL of pyrydine are added to the vial. The silylation reaction is conducted at 60 ºC for 30 min. After completion, the mixture is

diluted with EtOAc to 1 mL and subjected to the GC-MS/FID. The only observed peak corresponds to the internal standard and no monomers are observed (Fig. S17). Because thioacidolysis targets alkyl aryl ether linkages (including β–O–4, as the most abundant in hardwood lignins) to release monomers, the absence of monomer peaks indicates that no cleavable β–O–4 linkages remained in the residual lignin. This suggests that the remaining lignin underwent recondensation during the organocatalysis-first step.

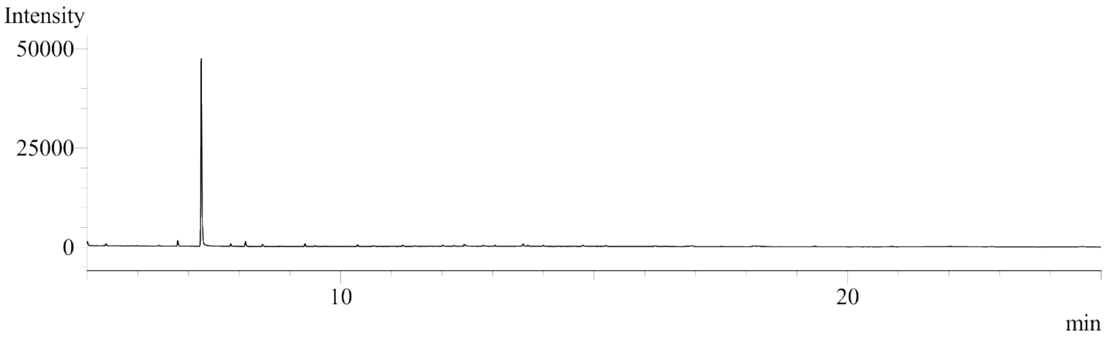

Figure S17. GC-FID of the thioacidolysis of the solid residue after organocatalysis.

References

(1) Talebi Amiri, M.; Dick, G. R.; Questell-Santiago, Y. M.; Luterbacher, J. S. Fractionation of Lignocellulosic Biomass to Produce Uncondensed Aldehyde-Stabilized Lignin. *Nat. Protoc.* **2019**, *14* (3), 921–954. https://doi.org/10.1038/s41596-018-0121-7.

(2) Holman, J. D.; Tabb, D. L.; Mallick, P. Employing ProteoWizard to Convert Raw Mass Spectrometry Data. *Curr. Protoc. Bioinforma.* **2014**, *46* (1), 13.24.1-13.24.9. https://doi.org/10.1002/0471250953.bi1324s46.

(3) Schmid, R.; Heuckeroth, S.; Korf, A.; Smirnov, A.; Myers, O.; Dyrlund, T. S.; Bushuiev, R.; Murray, K. J.; Hoffmann, N.; Lu, M.; Sarvepalli, A.; Zhang, Z.; Fleischauer, M.; Dührkop, K.; Wesner, M.; Hoogstra, S. J.; Rudt, E.; Mokshyna, O.; Brungs, C.; Ponomarov, K.; Mutabdžija, L.; Damiani, T.; Pudney, C. J.; Earll, M.; Helmer, P. O.; Fallon, T. R.; Schulze, T.; Rivas-Ubach, A.; Bilbao, A.; Richter, H.; Nothias, L.-F.; Wang, M.; Orešič, M.; Weng, J.-K.; Böcker, S.; Jeibmann, A.; Hayen, H.; Karst, U.; Dorrestein, P. C.; Petras, D.; Du, X.; Pluskal, T. Integrative Analysis of Multimodal Mass Spectrometry Data in MZmine 3. *Nat. Biotechnol.* **2023**, *41* (4), 447–449. https://doi.org/10.1038/s41587-023-01690-2.

(4) *SIRIUS 4: a rapid tool for turning tandem mass spectra into metabolite structure information | Nature Methods*. https://www.nature.com/articles/s41592-019-0344-8 (accessed 2026-03-19).
